# Dinuclear Group 4 Metallocene Catalysts of the Type [(Cp_2_M)_2_(*μ*‐Me)(*μ*‐C_2_R)]: Structure‐Activity Relationships in Ethylene Polymerization

**DOI:** 10.1002/chem.71125

**Published:** 2026-05-21

**Authors:** Hanan Al Hamwi, Mirko Rippke, Anke Spannenberg, Michal Horáček, Jiří Pinkas, Vojtěch Varga, Martin Lamač, Fabian Reiß, Torsten Beweries

**Affiliations:** ^1^ Leibniz Institute for Catalysis Rostock Germany; ^2^ J. Heyrovsky Institute of Physical Chemistry of the Czech Academy of Sciences Prague Czech Republic

**Keywords:** DFT, dinuclear complexes, ethylene polymerization, metallocenes, radicals

## Abstract

The heterodinuclear zirconocene/titanocene complexes, [Cp_2_Zr(μ‐Me)(μ‐C_2_R)(TiCp_2_)] where R = SiMe_3_ and R = Ph, were prepared using the previously reported comproportion reaction of the zirconocene alkynyl methyl complex with Rosenthal's zirconocene source. Examination of the molecular structure revealed alkynyl group migration from Zr to Ti, as confirmed by single‐crystal x‐ray analysis, nuclear magnetic resonance spectroscopy, and quantum chemical calculations. The homodinuclear (Zr/Zr) and heterodinuclear (Ti/Zr) complexes were activated with [Ph_3_C][B(C_6_F_5_)_4_], B(C_6_F_5_)_3_, and a mixture of B(C_6_F_5_)_3_ with excess Et_3_SiH (SiHB system) as aluminium‐free activators and tested in ethylene polymerization. Depending on the type of activator, unique reactivity was observed, resulting in either electron or methyl abstraction and the formation of cationic species. The catalytically active species are proposed to have dinuclear character, forming linear polyethylenes with unsaturated groups predominantly on the Zr cationic center. In contrast, the Ti cationic center generated by a SiHB system only in dichloromethane produced silicon‐terminated polyethylenes.

## Graphical abstract

Homo‐ and heterodinuclear group 4 metallocene complexes of the type [(Cp_2_M)_2_(μ‐Me)(μ‐C_2_R)] (M = Ti, Zr) are active catalysts for the polymerization of ethylene in the presence of aluminium‐free activators. The catalytically active species are proposed to have dinuclear character and depending on the type of activator, unique reactivity was observed, resulting in either radical or methyl abstraction and the formation of cationic species.

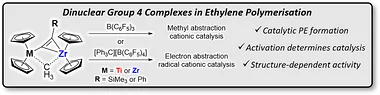

## 1 Introduction

Multinuclear transition metal complexes have emerged as powerful catalysts for the polymerisation of olefins due to their ability to promote cooperative effects between multiple metal centres [1]. In these systems, the proximity of two or more metal atoms can facilitate enhanced monomer activation, improved chain propagation rates, and greater control over polymer microstructure compared to mononuclear analogues [2]. The interplay between the metals — t hrough electronic communication or shared ligands — can stabilise key intermediates, leading to tunable activity and selectivity. Such complexes have been particularly valuable in developing catalysts for ethylene and propylene (co‐)polymerisation, where fine‐tuning the metal–metal distance and ligand environment allows for the precise design of materials with tailored molecular weights and stereoregularity [3].

**FIGURE 1:**
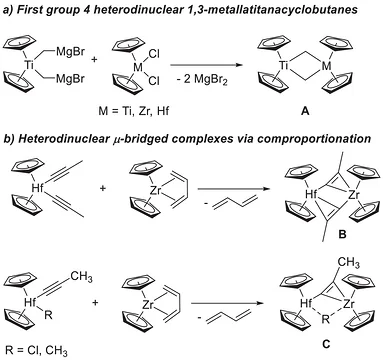
Selected literature‐known metallocene‐based heterodinuclear group 4 metal complexes.

Over the past years, a wide range of metallocene and non‐metallocene‐based group 4 dinuclear complexes have been investigated with regard to their catalytic activity in ethylene and olefin copolymerization [4]. Essentially, homodinuclear complexes possessing Ti_2_ [5, 6, 7, 8, 9], Zr_2_ [8, 9, 10, 11, 12, 13, 14], and Hf_2_ [15] were investigated, with most cases showing a significant increase in activity in the dinuclear complexes compared to the mononuclear analogues. In contrast, to the best of our knowledge, a very limited number of group 4 heterodinuclear complexes have been investigated and studied in terms of their catalytic activity in ethylene polymerisation [16, 17, 18]. In the above‐mentioned homodinuclear examples, both group 4 metals are usually introduced to the ligand in a single step, so that no hetero dinuclear complexes are accessible in this way. The first synthesis of heterodinuclear complexes of group 4 metals was reported in 1985 by Bickelhaupt and co‐workers using bimetallic di‐Grignard reagents in combination with metallocene dihalides, which form 1,3‐metallatitanacyclobutanes (**A**, Figure 1a) [19]. Later, Erker's research group discovered another modular synthesis approach via a comproportionation of two different zirconocene complexes in the oxidation states +2 and +4 [20]. This concept was further investigated to form a heterodinuclear group 4 complex in which zirconocene and hafnocene fragments are bridged via two (μ‐alkynyl) units (**B**, Figure 1b, top). The following development also shows how two different μ‐bridging units can be introduced using this approach. In this context, one of the alkynyl ligands was replaced by either a chloride or a methyl μ‐bridging unit (**C**, Figure 1b, bottom) [21].

In a previous study, some of us have used this redox comproportionation approach by replacing the zirconocene(II) butadiene reagent with the more convenient Rosenthal complex [Cp_2_Zr(py)(Me_3_SiC_2_SiMe_3_)] (**1**, py = pyridine) to access homodinuclear complexes with a structural motif similar to that of **C** (R = Me) [22, 23]. Having verified this methodology, we reasoned that changing from Rosenthal's Zr(II) source to the corresponding Ti(II) source (i.e., [Cp_2_Ti(Me_3_SiC_2_SiMe_3_)], **2**) would give us a modular access to a wide range of heterodinuclear complexes with different steric and electronic profiles. In this contribution we present the synthesis and characterisation of a set of homo‐ and heterodinuclear group 4 metallocene complexes. The suitability of these systems for ethylene polymerisation is evaluated in a series of experiments using different well‐known activators.

## 2 Results and Discussion

### 2.1 Synthesis of Dinuclear Precatalysts

**SCHEME 1:**
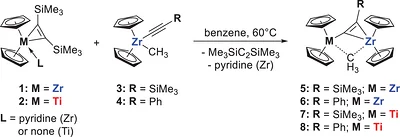
Synthesis of complexes **5–8** using zirconocene alkynyl complexes **3** and **4** in combination with Cp_2_M(II) reagents **1** and **2**.

In our previous work, we have synthesised the homodinuclear zirconocene complexes [(Cp_2_Zr)_2_(μ‐Me)(μ‐C_2_R)] (**5**: R = SiMe_3_ [22] and **6**: R = Ph [23]) in moderate yields by combining the alkynyl starting compounds **3** and **4** with the zirconocene(II) source **1** in benzene and heating to 60°C (Scheme 1). Using the same conditions, replacing the Zr(II) reagent by [Cp_2_Ti(II)(Me_3_SiC_2_SiMe_3_)] (**2**), we were now able to access two heterodinuclear zirconocene/titanocene complexes [(Cp_2_Zr)(μ‐Me)(μ‐C_2_R)(TiCp_2_)] (**7**: R = SiMe_3_; **8**: R = Ph) (Scheme 1). After stirring the benzene solutions at 60°C for 24 h, all volatile components were removed under vacuum and an attempt was made to dissolve the residues obtained in pentane, which showed that **7** is moderately soluble and **8** is less soluble. Consequently, a saturated solution of **7** in pentane was filtered and recrystallised at low temperature (–78°C), while **8** was washed with pentane. The solids obtained were isolated, yielding complexes **7** and **8** in rather poor yields of 23% and 33%, respectively. It should be noted that repeated attempts to optimize this procedure to obtain better yields resulted in significantly impure isolated solids.

**FIGURE 2:**
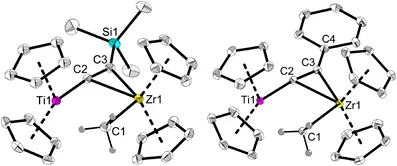
Molecular structures of complex **7** left and **8** right. Thermal ellipsoids correspond to 30% probability at 150 K. Hydrogen atoms except at C1 are omitted for clarity. Selected bond lengths are given in Table 1. Note: Complex **7** crystallizes with one molecule of toluene in the asymmetric unit. This molecule and one Cp ligand each of the titanocene and zirconocene units are disordered. For clarity, the minor part of disorder and solvent molecule has been omitted from the figure.

As expected, the ^1^H and ^13^C NMR spectra of the isolated heterodinuclear complexes **7** and **8** differ significantly from those of the homodinuclear analogues. For example, in **5** and **6**, the ^1^H NMR signals of the Cp units are barely distinguishable from each other, whereas in **7** and **8** these resonances are separated by 0.3 ppm. ^1^H NMR signals of the bridging methyl ligands are shifted to higher field (**7**: –4.12 ppm; **8**: –3.72 ppm), suggesting significant electronic differences compared to the mononuclear precursor complexes **3** and **4** (vide infra). Remarkably, the ^13^C{^1^H} NMR spectra showed the resonances of the α‐C atoms of the μ‐bridging alkynyl units shifted by 15–20 ppm to lower field (Table 1). In contrast, the β‐C resonances are significantly shielded, which indicates a different connectivity of the alkyne bridge compared to the homodinuclear compounds. To further confirm this, single crystals suitable for SC‐XRD analysis were grown by slowly cooling a toluene/pentane solution from room temperature to −70°C (for **7**) and by cooling a saturated benzene solution from 80°C to room temperature (for **8**). The molecular structures of **7** and **8** are depicted in Figure 2.

**TABLE 1:** Selected ^13^C NMR spectroscopic and structural parameters of the dinuclear complexes **5–8**.

Complex	α ^13^C (ppm)	β ^13^C (ppm)	C2‐C3 (Å)	C1‐M1^a^ (Å)	C1‐M2 (Å)
**5** [22]	259.5	180.9	1.277(2)	2.520(2)	2.474(2)
**6** [23]	247.2	186.5	1.298(6)	2.453(5)	2.580(5)
**7**	276.6	165.2	1.269(4)	2.493(3)	2.385(3)
**8**	267.4	170.5	1.281(5)	2.496(3)	2.429(3)

^a^ M1 refers to the metal center to which the alkynyl unit is σ bound.

When examining the resulting molecular structures, it becomes clear that, contrary to expectations, the alkynyl group is not bound to the zirconocene via a σ bond as in the starting materials, but rather to the titanocene, explaining the more pronounced low‐field shift of the α‐C signal in ^13^C{^1^H} NMR. However, this raises questions about possible dynamics of the bridging alkynyl unit in the heterodinuclear complexes. In previous work [22, 23, 24] it was shown that oscillation of the formal methylide group between the two metal centers has significantly lower energy barriers than the shift of the bridging alkynyl unit. Furthermore, it was demonstrated that, according to quantum mechanical calculations at the B3LYP [25, 26, 27, 28, 29]‐D3 [30, 31]/def2‐TZVP [32, 33] level, the thermodynamically most stable isomer can be well predicted: in accordance with the connectivity determined in the solid state, complex **5** can be best described as a formal Zr(III)‐Zr(III) complex and complex **6** as a Zr(IV)‐Zr(II) complex (Table 1).

**FIGURE 3:**
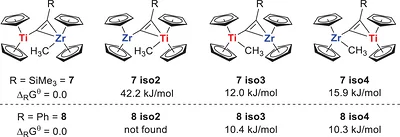
Thermodynamic data of four different possible isomers of complexes **7** and **8** (level of theory B3LYP‐D3/def2‐TZVP).

Motivated by this intriguing behavior, we calculated four possible isomers for compounds **7** and **8** at the same level of theory, considering the fluxionality of the methyl group and the alkynyl unit (Figure 3). Except for isomer **8 iso2**, the geometries could be optimized, and their energies were calculated. In the latter case, the **8 iso4** connectivity was consistently optimized as the final structure in several attempts, which is in accordance with the large energy difference between **7 iso2** and **7 iso4**, and a rather low energy barrier for the methyl group shift. The energy differences between the individual isomers of compounds **7** and **8** demonstrate that the connectivity determined in the solid state corresponds to the thermodynamically most stable form, explaining the rather unexpected alkynyl group shift from the zirconocene to titanocene during synthesis (Figure 3).

In order to shed more light on the electronic situation in complexes **7** and **8**, a series of different bond analyses were performed based on the optimised geometries. It was found that both complexes show RHF/UHF instability. The resulting broken symmetry solution shows two opposite spin densities at the metal centres in d_xy_ orbitals and in the p‐Cα orbital in the same plane, which indicates the presence of Ti(III) and Zr(III) centres, respectively (Figure S9 for **7**, Figure S12 for **8**). Although the formal oxidation states suggest a Ti(III)/Zr(III) d^1^–d^1^ system, the DFT results indicate a closed‐shell ground state arising from strong delocalization over the alkyynyl bridging unit. The HOMOs corresponds to a three‐centre bonding interaction (Figure S10 for **7**, Figure S13 for **8**), consistent with an alkynyl‐mediated metal–metal interaction explaining the antiferromagnetic coupling of the Ti(III) and Zr(III) centres and thus the diamagnetic properties of these compounds. This interpretation is supported by the absence of a QTAIM bond critical point between the metal centres in both cases, as well as the moderate Ti‐Zr Wiberg bond indices of 0.45 for **7** (Figures S8) and 0.43 for **8** (Figure S11), respectively. Furthermore, the pronounced high field shifts of the methyl ligands in the ^1^H NMR spectra (**7**: –4.12 ppm; **8**: –3.72 ppm) support the assumption of electron‐rich Zr(III) centres in both complexes, when compared to the Zr(IV) precursors (**3**: 0.18 ppm; **4**: 0.26 ppm).

Based on these findings, it appears that among the compounds investigated here compound **6** is the only species showing mixed‐valent behavior in the solid state. Any influence on its activity/reactivity is therefore of particular interest. Therefore, we decided to further investigate these four structurally related complexes as precatalysts in the polymerization of ethylene.

### 2.2 Polymerisation of Ethylene

Cationic alkyl (or hydride) complexes of group 4 metallocenes of the type [Cp_2_M(IV)R]^+^ are generally considered to be catalytically active in metallocene olefin polymerisation [2, 34, 35]. In view of this fact, we decided to test bimetallic complexes **5–8** and the zirconocene alkynyl precursors **3** and **4** in ethylene polymerization in the presence of aluminium‐free activators, namely [Ph_3_C][B(C_6_F_5_)_4_] (TBT), B(C_6_F_5_)_3_ and a mixture of B(C_6_F_5_)_3_ with excess of Et_3_SiH (SiHB system). Typically, the catalyst activation is initiated by the abstraction of one alkyl or hydrido group from the metallocene to form an ion‐pair complex. In the case of the studied bimetallic complexes, initial valence state of the metals as well as the reactivity of individual ligands seemed non‐intuitive.

#### 2.2.1 Polymerisation Reactions With [Ph_3_C][B(C_6_F_5_)_4_] (TBT) as the Activator

Results of ethylene polymerisation using the bimetallic complexes **5–8**, the precursors **3** and **4**, and the reference compound Cp_2_ZrMe_2_ activated with TBT are shown in Table 2. The activity of bimetallic complexes/TBT depended predominantly on the nature of the bridging acetylide ligand rather than on the nature of metals. Compounds **6** and **8**, which contain phenylacetylide bridging units, exhibit very high activity levels of up to 8,740 kg_PE_ (mol_cat_ bar h)^−1^, comparable to those of the reference catalyst Cp_2_ZrMe_2_ [36]. Contrary to that, the trimethylsilylacetylide counterparts demonstrate either one order of magnitude lower activity (**7**) or negligible activity (**5**) under the same conditions, respectively (Table 2). Remarkably, only trace amounts of polyethylene (PE) were obtained using mononuclear precursors **3** and **4** under the same conditions, highlighting the importance of the dinuclear motif for catalytic activity using this activator.

**TABLE 2:** Ethylene polymerization initiated by the systems “precatalyst”/TBT.^a^

Precat.	*t* (s)	*T*_2_ (°C)	*PE* (g)	*A* ^b^	*M*_n_ (g/mol)	*M*_w_/*M*_n_
**5**	613	25	*trace*	*trace*	*n.d*.	*n.d*.
**6**	55	39	0.885	7,720	196,400	3.25
**7**	314	29	0.325	500	139,100	3.01
**8**	48	39	0.874	8,740	138,500	3.76
**3**	339	25	*trace*	*trace*	*n.d*.	*n.d*.
**4**	315	25	*trace*	*trace*	*n.d*.	*n.d*.
Cp_2_ZrMe_2_	56	40	1.083	9,280	140,200	4.13

^a^ *Polymerization conditions*: total volume 50 mL, polymerization medium toluene, precatalyst dissolved in toluene, pressure 1 bar, T_1_ = starting temperature 25^°^C, T_2_ = final temperature just before quenching, rpm 800, [precatalyst] = 7.5 µmol L^–^^1^, TBT/precatalyst = 1.1.

^b^ A = kg_PE_ (mol_cat_ bar h)^−1^.

All of the polymers obtained in the experiments presented in Table 2 are linear PEs with unsaturated end groups, such as vinyl and internal vinylidene groups (as detected by IR spectroscopy ‐ See Figures S40, S41). According to gel permeation chromatography (GPC) analysis, they exhibit broad dispersity, similar to samples produced using the reference complex Cp_2_ZrMe_2_, in line with the exothermic behaviour of these catalytic runs. The increase in molar mass dispersity can be attributed to enhanced β‐hydride elimination processes at elevated temperatures, which were difficult to control under the selected conditions for highly active systems (for more details about the polymerisation procedures see the Supporting Information, section 5).

The polymerisation mixtures changed colour immediately after injection of the metallocene complex to the activator solution to blue (**5**/TBT system) or green (for **6** and **7**/TBT systems), which suggests changes in the oxidation state of the involved metal centres. Therefore, an EPR investigation was performed (discussed below).

TBT activation of monometallic precursor complexes (**3** and **4**) led to systems possessing only negligible activity. However, a small amount of PE was obtained when the concentration of **4**/TBT was doubled (see Table S8).

#### 2.2.2 Polymerisation Reactions With B(C_6_F_5_)_3_ and B(C_6_F_5_)_3_/Et_3_SiH as the Activators

The results of the polymerisation tests involving bimetallic complexes **5–8**, precursors **3** and **4**, and reference compounds upon activation with the Lewis acid B(C_6_F_5_)_3_ are presented in Table 3.

**TABLE 3:** Ethylene polymerization initiated by the systems “precatalyst”/B(C_6_F_5_)_3_.^a^

Precat.	*t* (s)	*T*_2_ (°C)	*PE* (g)	*A* ^b^	*M*_n_ (g/mol)	*M*_w_/*M*_n_
**5**	201	29	0.340	140	103,000	3.31
**6**	198	42	1.692	680	159,000	2.95
**7**	76	45	1.803	1,900	141,000	3.28
**8**	85	43	1.240	1,170	134,900	3.29
**3**	480	25	*trace*	*trace*	*n.d*.	*n.d*.
**4**	365	28	0.210	50	93,000	4.66
Cp_2_ZrMe_2_	41	56	2.034	3,970	107,300	3.15
Cp_2_TiMe_2_	610	34	0.220	30	15,700	11.9^c^

^a^ *Polymerization conditions*: total volume 50 mL, polymerization medium toluene, precatalyst dissolved in toluene, pressure 3 bar, T_1_ = starting temperature 25°C, T_2_ = final temperature before quenching, rpm 800, [precatalyst] = 15 µmol L^–^^1^, B(C_6_F_5_)_3_/precatalyst = 1.1.

^b^A = kg_PE_ (mol_cat_ bar h)^−1^.

^c^Bimodal M_w_/M_n_ distribution (see Figure S48).

In general, these systems were less active than the TBT system, while the Ti containing species **7** and **8** showed approximately ten− and two−times higher activity in comparison to the corresponding zirconium analogues **5** and **6**, producing polymers with similar molecular weights and dispersities. Monometallic precursors **3** and **4** were again almost inactive. We may speculate that the modest activity of **4**/B(C_6_F_5_)_3_ system could arise from a partial dissociation of the proposed dimeric dication [Cp_2_Zr(C_2_R)]_2_^2+^ by coordination of the MeB(C_6_F_5_)_3_^−^ anion. In contrast to the TBT system, a gradual increase in the ethylene consumption in the course of polymerisation was observed for the activation with B(C_6_F_5_)_3_ and a similar increase (up to the reactor fouling) was also observed for the SiHB system (Table 4). This contrasts with the different kinetics observed for the SiHB system activation of metal‐halogen bond‐containing precatalysts in our previous studies, where a hydride transfer process occurred from the silane to the metals [37, 38, 39, 40, 41]. This suggests a different role of the silane in the studied systems, where it can act only as a chain‐transfer agent.

**TABLE 4:** Ethylene polymerization initiated by the systems “precatalyst”/B(C_6_F_5_)_3_ in the presence of Et_3_SiH.^a^

Entry	Precat.	Polym. medium	*t* (s)	*T*_2_ (°C)	*PE* (g)	*A* ^b^	Si/1000C^c^	Si‐terminated^d^ (%)	*M*_n_ (g/mol)	*M*_w_/*M*_n_
1	**5**	toluene	500	30	0.810	130	—	—	147,700	2.81
2	**6**	toluene	198	53	2.410	970	—	—	112,700	3.45
3	**6**	CH_2_Cl_2_	103	54	2.033	1,580	—	—	75,000	3.43
4	**7**	toluene	197	44	1.934	790	—	—	71,300	4.83
5	**7**	CH_2_Cl_2_	560	28	0.555	80	1.416	86	8,500	3.67
6	**8**	toluene	76	62	3.174	3,340	—	—	36,500	8.43
7	**8**	CH_2_Cl_2_	317	46	1.915	480	2.624	84	4,500	3.14
8	**8** ^e^	CH_2_Cl_2_	55	44	1.256	1,830	2.441	64	3,700	3.26
9	Cp_2_ZrMe_2_	toluene	35	74	2.757	6,300	—	—	21,000	6.14
10	Cp_2_TiMe_2_	toluene	31	63	1.715	4,430	5.047	76	2,100	2.6
11	Cp_2_TiMe_2_	CH_2_Cl_2_	36	52	1.318	2,930	4.353	75	2,400	3.0
12	Cp_2_TiCl_2_	toluene	1800	*n.d*.	0.074	3	0.19	11	8,200	*n.d*.
13	Cp_2_TiCl_2_	CH_2_Cl_2_	120	*n.d*.	1.570	1,050	2.88	92	4,500	*n.d*.

^a^ *Polymerization conditions*: total volume 50 mL, catalyst dissolved in toluene, pressure 3 bar, T_1_ = starting temperature 25°C, T_2_ = final temperature before quenching, rpm 800, [precatalyst] = 15 µmol L^–^^1^, Et_3_SiH/precat. = 1,000, B(C_6_F_5_)_3_/precat. = 1.1.

^b^A = kg_PE_ (mol_cat_ bar h)^−1^.

^c^Number of Si atoms per 1000 C atoms in the polymer (determined by FT‐IR).

^d^Percentage of silyl‐terminated polymer chains as determined by FT‐IR.

^e^4.3 mL of CH_2_Cl_2_ was added to the rest of catalyst solution (in toluene) prepared for entry 6 and kept 24 h at room temperature under argon.

Indeed, polymerisation reactions in toluene upon the SiHB activation showed similar activity of the catalysts as in the absence of Et_3_SiH (Table 4). Most notably, the presence of silane in the reaction mixture appeared crucial in terms of the resulting polymer's properties when the polymerisation medium was changed from toluene to dichloromethane. The use of the Ti‐containing bimetallic complexes **7** and **8** resulted in lower catalytic activity and molecular weight but yielded highly silyl‐capped polyethylene (SiPE) in dichloromethane (entry 5, 7 and 8 in Table 4). Notably, the same PE samples exhibited only a negligible amount of unsaturated chain ends. This indicates that polymerisation on the zirconocene part is suppressed under these conditions. High activity associated with the production of low‐molecular‐weight SiPE polymers was also observed for the reference substance, Cp_2_TiMe_2_ (entry 10 and 11 in Table 4).

#### 2.2.3 EPR/NMR Analysis of In Situ Activation in Absence of Ethylene

EPR analyses of the reaction mixtures of complexes **5–8** with TBT as an activator were performed in view of the potential presence of paramagnetic d^1^ zirconocene or titanocene fragments and organic radical species. All the reactions studied in toluene solutions resulted in the immediate formation of the trityl radical (Ph_3_C^•^). This radical is characterised by a multiplet signal at a g‐value of 2.003. Hyperfine splitting of the signal, caused by coupling of the unpaired electron with hydrogen atoms on the phenyl rings, was a_H_ = 0.12 mT. The reactions produce partially insoluble oily products and are accompanied by a colour change from yellow to turquoise or green (similarly as during the initial phase of polymerisation), indicating the formation of cationic and/or paramagnetic Zr or Ti species, as well as radicals. In the case of complexes **5** and **6**, which only contain Zr atoms, paramagnetic, trivalent Zr species with a g‐value of 1.997 and ^91^Zr (5/2) splitting a_Zr_ = 1.4 mT are formed once all the components have dissolved, resulting in a green oil which is sparingly soluble in toluene. The stability of paramagnetic species varies considerably. Complex **6** exhibits a faster decrease in the paramagnetic signal intensity than complex **5**, with the latter remaining visible even after 24 hours. Heteronuclear complexes **7** and **8** also form paramagnetic species after TBT activation and a g‐factor value more typical of Ti(III) paramagnetic species (1.977).

NMR spectroscopy of the above reactions of complexes **5–8** with TBT revealed the presence of Gomberg´s dimer (Ph_2_C = C_6_H_5_CPh_3_) [42], which is a known product of the Ph_3_C^•^ radical dimerization [43, 44], in the toluene soluble fraction (Figure S61). In addition, an absence of Ph_3_CMe in these samples suggested that the methyl group has not been abstracted in a similar way as for complex **4** (see below). Observation of the major metal‐containing products of these reactions was hampered by their inherent paramagnetism and low solubility, which is also indicative of their ionic nature. We attempted to study these compounds after mild oxidation by dissolving the oils from reactions of complexes **7** and **8** in CDCl_3_ (a small amount of THF was typically added to stabilize the potential cationic fragments). NMR spectra (Figures S62, S63) revealed the presence of the expected [B(C_6_F_5_)_4_]^–^ anion and several metallocene species among which we tentatively identified the [Cp_2_TiCl(THF)]^+^ cation, which we subsequently prepared in a separate experiment by chloride abstraction from Cp_2_TiCl_2_ with [Et_3_Si][B(C_6_F_5_)_4_] in the presence of THF (see the Supporting Information for details). To the best of our knowledge, this cationic complex has not been previously reported, but similar cationic species of the formula [Cp_2_TiCl(L)]^+^ (L = H_2_O, nitrile, etc.) are known [45, 46, 47, 48, 49].

NMR investigation of **4**/TBT mixture, in contrast to complexes **5–8**, revealed the presence of Ph_3_CMe (Figure S60), which indicated the expected methyl abstraction and formation of cationic species of the formula [Cp_2_Zr(C_2_SiMe_3_)]^+^. We propose that this strongly electrophilic species undergoes dimerization similarly as it is known for the corresponding paramagnetic group 4 monoacetylides [20, 24, 50, 51, 52, 53], which effectively precludes ethylene polymerisation. In addition, traces of Gomberg's dimer were also observed in the spectra, which was confirmed by subsequent EPR measurements (Figure S58). Additional control EPR experiments excluded the formation of Ph_3_C^•^ radical in pure toluene TBT solutions. However, mixing Cp_2_ZrMe_2_ with TBT in toluene also resulted in the observation of the sole trityl radical signal (Figure S57). Importantly, no mononuclear Zr(III) species were observed in reactions of **4** and Cp_2_ZrMe_2_ with TBT. In addition, EPR measurements of the neutral compound **5** in toluene showed no signal, even at low temperatures in a toluene glass (−160°C).

The reactions of complexes **5–8** with B(C_6_F_5_)_3_ in toluene also produced oily precipitates, which complicated a direct observation of the in situ formed products. EPR revealed the presence of several unidentified Ti(III) species (g‐value of the main signal 1.970) in the reaction mixture of **8** (see Figure S52). NMR measurements on the sample of oil from the reaction of **8** with B(C_6_F_5_)_3_ dissolved in CDCl_3_ (used to fully oxidize low valent species) confirmed the presence of [MeB(C_6_F_5_)_3_]^–^ anion (Figure S62d and S64, S65), which clearly indicated the previous methyl group abstraction from the original complex. Interestingly, the ^1^H NMR spectra featured the same signal for [Cp_2_TiCl(THF)]^+^ as described above, when THF was introduced to the mixture, and also a smaller amount of Cp_2_TiCl_2_. Signals attributable to the Cp_2_Zr‐alkynyl fragment could also be observed, although not unambiguously assigned.

In the context of previous experiments, pure complex **8** was also analysed by NMR in CDCl_3_ solution (Figure S66), which revealed its instability in this solvent, predominantly giving rise to Cp_2_Zr(Me)Cl and several other species, of which Cp_2_TiCl_2_ can be identified. This is in line with the structural description presented above featuring the “Cp_2_ZrMe” fragment.

#### 2.2.4 Computational Analysis of Precatalyst Activation

**SCHEME 2:**
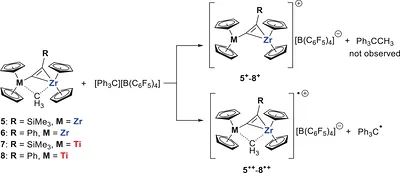
Investigation of different activation sequences of complexes **5–8**, and TBT. Level of theory B3LYP‐D3/def2‐TZVP.

Computational analysis was done for the plausible activation sequence with TBT, yielding either cationic species **5** ^+^–**8** ^+^ (relevant to the methyl‐abstraction route with B(C_6_F_5_)_3_) or the radical cations **5** ^•+^–**8** ^•+^ in two distinct reaction pathways (Scheme 2, Table 5). In all cases this shows highly exergonic formation of both species. Thus, both reaction channels are theoretically possible in parallel.

**TABLE 5:** Computed thermodynamic values for the formation of the cationic species **5** ^+^–**8** ^+^ and the radical cations **5** ^•+^–**8** ^•+^, and the summarized natural charges and spin densities at the individual Cp_2_M1 and Cp_2_M2 fragments.

Complex	Δ_R_H^Ө^ (kJ·mol^−1^)	Δ_R_G^Ө^ (kJ·mol^−1^)	∑q_(Nat.NBO)_ Cp_2_M1^a^	∑q_(Nat.NBO)_ Cp_2_M2^a^
**5^+^**	−50.2	−65.9	0.968	0.973
**5^•+^**	−65.8	−73.0	1.055/*0.27*	1.021/*0.31*
**6^+^**	−78.1	−85.3	0.940	0.870
**6^•+^**	−52.5	−60.6	1.098/*0.22*	1.057/*0.30*
**7^+^**	−77.5	−93.8	0.703	1.071
**7^•+^**	−59.1	−68.4	0.731/*0.50*	1.092/*0.21*
**8^+^**	−100.3	−110.7	0.596	0.971
**8^•+^**	−53.6	−55.5	0.752/*0.43*	1.089/*0.23*

^a^ second value in italics represents the sum of natural spin in the radical cationic species.

Only in the case of **5** featuring two Zr centres and a SiMe_3_ substituent at the alkynyl unit the formation of the radical cation **5^•+^** is slightly favoured, which could explain the longer stability of the observed EPR‐active species (vide supra). Nevertheless, we found no experimental evidence for the formation of the cationic species **5^+^**–**8^+^**, which may be due to high activation barriers which we did not further investigate here.

**FIGURE 4:**
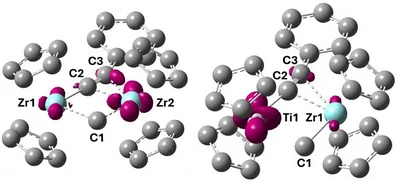
Representation of the spin density plots of radical cations **6** ^•+^ (left) and **8** ^•+^ (right). Hydrogen atoms were omitted for clarity. Level of theory: B3LYP‐D3/def2‐TZVP.

In addition to these considerations, we examined the spin density plots of the calculated radical cations **5^•+^**–**8^•+^** to gain further insights into the different activities of these species in the polymerisation process. In case of the heterodinuclear complexes **7** and **8**, the spin densities are mainly localised at the titanocene centre, which is σ‐bonded to the alkynyl unit (Figure 4, right and Figure S40), while in the case of homodinuclear complexes **5** and **6**, the spin densities are more evenly distributed but show larger contributions at the zirconocene centres that are π‐bonded to the alkynyl unit (Figure 4, left and Figure S38).

These findings are well supported by additionally performed NBO [54, 55, 56, 57] analysis and their sum of natural spin in the individual Cp_2_M1/2 fragments (Table 5). Furthermore, we evaluated the summed natural charge distribution at the individual metallocene fragments Cp_2_M1 and Cp_2_M2 (Table 5), which show more balanced charges in the homodinuclear cationic species (**5^+^** and **6^+^**) compared to the heterodinuclear cationic species (**7^+^** and **8^+^**) in which the zirconocene fragment shows the higher positive natural charge. Of note, only for the cations of **6** (i.e. **6^+^** and **6^•+^**) a different charge distribution was found compared to all other species in line with its different preferred connectivity as mixed‐valence species. In this case the zirconocene center (Cp_2_M1), which is σ‐bonded to the alkynyl unit shows the higher positive charge. An additional Wiberg bond index (WBI) analysis of these hypothetical activated species (Table S6) shows significantly larger M1‐Cα WBIs in the cationic species compared to the radical cationic species. Furthermore, it was found that the metal‐carbon WBIs between the alkynyl carbons (Cα and Cβ) and zirconium of the second metallocene fragment Cp_2_M2 in **5^•+^**–**8^•+^** are lower and the WBIs of the Cα‐Cβ bonds in these units are larger than in the cationic **5^+^–8^+^** species. Therefore, it can be assumed that the interaction between the alkynyl unit and the Cp_2_M2 fragment is weaker in the radical cationic species. Considering these results, we speculate that under catalytic reaction conditions the radical cationic species can dissociate into individual fragments. Based on the natural charge and spin distributions formation of [Cp_2_TiCCR]**^•^** and [Cp_2_ZrMe]^+^ could occur for the heterodinuclear complexes.

## 3 Discussion

**SCHEME 3:**
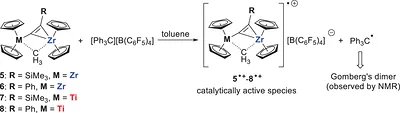
Proposed activation of complexes **5–8** using [Ph_3_C][B(C_6_F_5_)_4_] (TBT).

Based on the theoretical calculations and spectroscopic studies showing the presence of the Gomberg´s dimer and the corresponding Ph_3_C^.^ radical and absence of Ph_3_CMe in all samples after the reaction with TBT we propose that the activation of **5–8** with TBT proceeds as a redox reaction as depicted in Scheme 3 giving rise to a bimetallic radical cation, which contains the [Cp_2_ZrMe]^+^ cationic center with a coordinated Cp_2_M‐alkynyl radical moiety. In this case, the substituent on the alkynyl blocking the catalytic center seems to play a decisive role in the overall catalytic activity. Coordination of various alkynes to cationic zirconocene polymerization catalysts has previously been studied by Jordan et al. [58]. Subsequent dissociation of the dinuclear complex could release the catalytically active [Cp_2_ZrMe]^+^ and an inactive paramagnetic part. In line with this assumption, catalytic activities of these systems resemble that of the mononuclear complex Cp_2_ZrMe_2_. We suggest that the persistence of the paramagnetic dinuclear species **5^•+^** may be responsible for the low efficiency of **5** when activated with TBT.

**SCHEME 4:**
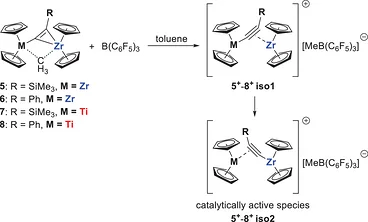
Proposed activation of complexes **5–8** using B(C_6_F_5_)_3_.

In contrast, precatalyst activation with B(C_6_F_5_)_3_ led to clean methyl abstraction from the dinuclear complex, which likely resulted in its rearrangement to give a catalytically active cationic Zr(IV) alkynyl species having the alkynyl fragment coordinated to the second metal center, likely in the +2 oxidation state (Scheme 4). This assumption is further supported by the computed low energy differences between the two resulting isomers of **7^+^** and **8^+^**. **7^+^ iso2** is computed to be slightly higher in energy (18 kJ·mol^−1^) than **7^+^ iso1**. In contrast, **8^+^ iso2** and **8^+^ iso1** are almost thermoneutral with a slight preference for **8^+^ iso2** by 1 kJ·mol^−1^. This might provide an explanation for the higher catalytic activity of **8** compared to **7** in the cationic regime. We furthermore propose that this additional coordination prevents dimerization of the cationic alkynyl complexes and thus contributes to an improved activity of the dinuclear complexes in comparison to monometallic systems such as those generated from **3** and **4**.

In the presence of Et_3_SiH, the system behaved similarly to activation using B(C_6_F_5_)_3_ alone, consistent with the absence of halide ligands on the metal. However, a fundamental change occurs when the polymerization solvent is changed to dichloromethane, which is known to oxidize and halogenate low‐valent titanocene complexes (vide supra). The oxidation of the low‐valent titanocene component of bimetallic complexes in **7** and **8** using dichloromethane resulted in lower polymerization activities and reduced molecular weights but yielded highly silyl‐capped polyethylene (SiPE). The production of these types of SiPE using titanocene dichloride after SiHB activation and the role of the oxidizing solvent are the subject of a recent patent [59]. The formation of SiPE via cationic titanocene centers after the SiHB activation is the subject of ongoing research.

## 4 Conclusion

Multinuclear complexes offer the possibility of adjusting the cooperative effects between several metal centres by selecting suitable ligand systems and bridge motifs, thereby tuning their reactivity and catalytic activity. Building on previous results, we report the synthesis and characterisation of two heterodinuclear group 4 metallocene alkynyl‐methyl complexes and its application as precatalysts for the polymerisation of ethylene in combination with aluminium‐free activators. Using a combination of structural, NMR, and DFT analysis both new complexes [Cp_2_Zr(μ‐Me)(μ‐C_2_R)(TiCp_2_)] (R = SiMe_3_, Ph) were characterised as Ti(III)/Zr(III) complexes, in contrast to a previously reported analogous mixed‐valent dinuclear Zr(II)/Zr(IV) complex bearing a phenylalkynyl ligand.

Activation of the dinuclear precatalysts using trityl borate is proposed to produce a dinuclear radical cation, which releases a catalytically active Zr cation that produces high‐molecular weight polyethylenes. Use of the Lewis acid B(C_6_F_5_) results in methyl abstraction from the dinuclear complex, yielding a catalytically active dinuclear cation with a fluxional alkynyl ligand that produces high‐molecular weight polymers. The corresponding Ti‐based systems display activities comparable to well‐known mononuclear Zr systems. Addition of silane as an additional activator to B(C_6_F_5_) does not affect activity of the Zr‐based systems but allows for the synthesis of highly silyl‐capped polyethylene when using Ti‐based precatalysts in halide‐containing solvent.

The present work highlights the potential of these highly modular dinuclear group 4 metallocene complexes for olefin polymerisation catalysis and shines light on structure‐activity relationships in these systems. These insights will be useful for the realisation and interpretation of new catalytic activity of this interesting class of organometallic compounds.

## Conflicts of Interest

The authors declare no conflicts of interest.

## Supporting information



The authors have cited additional references within the Supporting Information [60, 61, 62, 63, 64, 65, 66, 67, 68, 69, 70, 71, 72].

## Data Availability

The data that support the findings of this study are available from the corresponding author upon reasonable request.
