# Supplementary material for: Dinuclear Group 4 Metallocene Catalysts of the Type [(Cp2M)2(μ‐Me)(μ‐C2R)]: Structure‐Activity Relationships in Ethylene Polymerization
Source: Chemistry. 2026 May 21;32(33):e71125. doi: 10.1002/chem.71125 (PMC13532123; doi:10.1002/chem.71125)
Supplement: Supplementary file 1 [file CHEM-32-e71125-s001.docx]

Supporting Information

**Dinuclear Group 4 Metallocene Catalysts of the Type
[(Cp_2_M)_2_(*μ*-Me)(*μ*-C_2_R)]: Structure-Activity Relationships in Ethylene Polymerisation**

Hanan Al Hamwi^a^, Mirko Rippke^a^, Anke Spannenberg^a^, Michal Horáček*^b^_,_ Jiří Pinkas^b^, Vojtěch Varga^b^, Martin Lamač*^b^, Fabian Reiß*^a^ and Torsten Beweries*^a^

^a^ Leibniz-Institut für Katalyse e.V., Albert-Einstein-Str. 29a, 18059 Rostock, Germany

Email: fabian.reiss@catalysis.de, torsten.beweries@catalysis.de

^b^ J. Heyrovsky Institute of Physical Chemistry of the Czech Academy of Sciences, Dolejškova 2155/3, 182 23 Prague 8, Czech Republic

Email: michal.horacek@jh-inst.cas.cz, martin.lamac@jh-inst.cas.cz

**Table of Contents**

[1	Experimental details	S2](#_Toc223606871)

[2	Crystallographic details	S4](#_Toc223606872)

[3	Synthesis of compounds	S23](#_Toc223606873)

[4	Computational details	S29](#_Toc223606874)

[5	Ethylene polymerisation experiments	S68](#_Toc223606875)

[6	EPR experiments	S77](#_Toc223606876)

[7	NMR experiments	S83](#_Toc223606877)

[8	References	S87](#_Toc223606878)

# 1  Experimental details

## 1.1  General information

All manipulations were carried out in an oxygen- and moisture-free argon atmosphere using standard Schlenk and drybox techniques. The solvents were purified with the Grubbs-type column system “Pure Solv MD-5” and dispensed into thick-walled glass Schlenk bombs equipped with Young-type Teflon valve stopcocks. Cp_2_ZrCl_2_ (Aldrich, 98%) was stored under argon and used as received. Cp_2_Zr(Me)(C_2_Ph),^[1]^ Cp_2_Zr(Me)(C_2_SiMe_3_),^[2]^ Cp_2_Ti(η^2^-Me_3_SiC_2_SiMe_3_)^[3]^ and Cp_2_Zr(py)(η^2^-Me_3_SiC_2_SiMe_3_)^[4]^ were prepared according to literature procedures. Argon (Ar) (Linde, 99.999%) was used without further purification or drying. Polymerisation grade ethylene (SIAD, Czech Republic) and argon (5.0, Messer) for catalytic experiments were further purified by passing through a column filled with activated molecular sieves (5 Å) and an oxygen purifier equipment filled with a copper-based catalyst (CRS Oxygen Purifier, Model 1000, Aldrich).

**NMR spectra** were determined on Bruker AV300, AV400, or Avance NEO 500 instruments. ^1^H and ^13^C{^1^H} chemical shifts were referenced to the solvent signal: benzene-*d*_6_ (*δ*_H_ 7.16 ppm, *δ*_C_ 128.06 ppm), toluene-*d*_8_ (*δ*_H_ 2.08 ppm, *δ*_C_ 20.43 ppm), CDCl_3_ (*δ*_H_ 7.26 ppm, *δ*_C_ 77.16 ppm).^[5]^

**IR spectra** for characterisation of isolated precatalysts **7** and **8** were recorded on a Bruker Alpha FT-IR, ATR Spectrometer, spectra are not corrected. General information about IR characterisation of the resulting polyethylenes are given in chapter 5.1.1. of this document.

**MS analysis** was done using a Finnigan MAT 95-XP instrument (Thermo-Electron) in CI^+^/CI^-^ mode (isobutene).

**Melting points** are uncorrected and were determined in sealed capillaries under Ar atmosphere using a Mettler-Toledo MP 70.

**X-Ray diffraction data** were collected on a Bruker Kappa APEX II Duo diffractometer The structures were solved by direct methods (SHELXS-97)^[6]^ and refined by full-matrix least-squares procedures on *F*^2^ (SHELXL).^[7]^ Diamond^[8]^ was used for graphical representations.

**CHN analysis** was using a Leco TruSpec elemental analyser. At this point it should be pointed out that we could not obtain satisfactory elemental analysis for complexes **7** and **8**. Despite repeated recrystallisation, repeated measurements with and without oxidiser V_2_O_5_ and modified furnace temperature, we observed up to 20% less carbon content than calculated/expected. This observation is consistent with problems occurring for the previously published complexes of this class of dinuclear early transition metal complexes. This behaviour might be explained by formation of mixed zirconium/titanium-silicon-carbides (ceramics) in the furnace and therefore the carbon content dramatically decreases.^[9]^

**All DFT calculations** were carried out with the Gaussian 16^[10]^ package of molecular orbital programmes. Further details are given in the computational details section.

**EPR** spectra were recorded on a CW X-band EPR spectrometer MiniScope MS400 (Magnettech) equipped with microwave frequency counter FC 400 and temperature controller TC H03.

EPR spectrometer MiniScope MS400 (Magnettech) is an instrument with high measurement sensitivity 8*109 Spins/0,1 mT and magnetic field stability 1,5 μT/min. Microwave power range standard 100 μW – 50 mW. Ultra-fast automatic frequency control (AFC). Allows irradiation of samples in the resonator during the measurement using a parallel beam. Microwave frequency counter model FC400. Further Details regarding the performed EPR experiments are given in the corresponding section below.

**Polymerisations and polymer characterisation** details are in presented the corresponding sections below.

# 2 Crystallographic details

**Table S1.** Crystallographic details of complexes **7** and **8**.

| Compound | **7** | **8** |
| --- | --- | --- |
| Chem. Formula | C_33_H_40_SiTiZr | C_29_H_28_TiZr |
| Formula weight [g/mol] | 603.86 | 515.63 |
| Colour | brown | orange |
| Crystal system | monoclinic | monoclinic |
| Space group | *P*2_1_/*n* | *P*2_1_ |
| *a* [Å] | 8.2796(2) | 8.1100(5) |
| *b* [Å] | 25.5403(5) | 8.4087(5) |
| *c* [Å] | 14.1700(4) | 16.329(1) |
| *α* [°] | 90 | 90 |
| *β* [°] | 99.8361(9) | 93.5765(16) |
| *γ* [°] | 90 | 90 |
| *V* [Å^3^] | 2952.39(12) | 1111.38(12) |
| Z | 4 | 2 |
| *ρ*_calcd._ [g/cm^3^] | 1.359 | 1.541 |
| *μ* [mm^−1^] | 5.712 | 6.987 |
| *T* [K] | 150(2) | 150(2) |
| Radiation type | CuKα | CuKα |
| Measured reflections | 39467 | 13519 |
| Independent reflections | 5384 | 3913 |
| Reflections with *I* > 2*σ*(*I*) | 4866 | 3890 |
| *R*_int_ | 0.0512 | 0.0288 |
| *F*(000) | 1256 | 528 |
| *R*_1_(*R*[*F*^2^>2*σ*(*F*^2^)]) | 0.0354 | 0.0200 |
| w*R*_2_(*F*^2^) | 0.0967 | 0.0519 |
| GooF | 1.036 | 1.049 |
| No. of Parameters | 308 | 292 |
| CCDC | 2487237 | 2487238 |

## 2.1 Crystallographic details of complex 7

Hydrogen atoms attached to C1 could be found from the difference Fourier map and were refined freely. Furthermore, two Cp rings and the solvent molecule are disordered with occupancies of 0.574(7): 0.426(7) for C7A-C11A/C7B-C11B (Cp ring attached to Ti1), 0.626(9): 0.374(9) for C17A-C21A/C17B-C21B (Cp ring attached to Zr1) and 0.753(4): 0.247(4) for C27A-C33A/C27B-C33B (toluene). AFIX 59 1.42 for Cp and AFIX 66 for the six-membered ring of the toluene were used to optimise the geometry of these disordered fragments. Additionally, SIMU, DELU and EADP instructions were included to equalise the displacement parameters of the carbon atoms of the idealized groups (EADP for C7A-C11A, C7B-C11B; EADP for C27A-C33A, C27B-C33B; SIMU and DELU for C17A-C21A, C17B-C21B. The DFIX command was applied to the bond lengths C27A-C33A and C27B-C33B within the disordered toluene.

**Figure S1.** Labeling scheme of the molecular structure of **7**. Displacement ellipsoids correspond to 30% probability. Hydrogen atoms are omitted for clarity. Lower occupied parts of disorder are shown with unfilled lines.

**Table S2.** Bond lengths [Å] and angles [°] for complex **7**.

| C(1)-Zr(1) | 2.385(3) | C(7A)-H(7A) | 0.9500 |
| --- | --- | --- | --- |
| C(1)-Ti(1) | 2.494(3) | C(8A)-C(9A) | 1.409(3) |
| C(1)-H(1A) | 0.98(4) | C(8A)-H(8A) | 0.9500 |
| C(1)-H(1B) | 0.99(4) | C(9A)-C(10A) | 1.409(3) |
| C(1)-H(1C) | 0.99(4) | C(9A)-H(9A) | 0.9500 |
| C(2)-C(3) | 1.270(4) | C(10A)-C(11A) | 1.409(3) |
| C(2)-Ti(1) | 2.018(3) | C(10A)-H(10A) | 0.9500 |
| C(2)-Zr(1) | 2.355(3) | C(11A)-H(11A) | 0.9500 |
| C(3)-Si(1) | 1.840(3) | C(7B)-C(11B) | 1.409(4) |
| C(3)-Zr(1) | 2.342(3) | C(7B)-C(8B) | 1.409(4) |
| C(4)-Si(1) | 1.866(4) | C(7B)-H(7B) | 0.9500 |
| C(4)-H(4A) | 0.9800 | C(8B)-C(9B) | 1.409(4) |
| C(4)-H(4B) | 0.9800 | C(8B)-H(8B) | 0.9500 |
| C(4)-H(4C) | 0.9800 | C(9B)-C(10B) | 1.409(4) |
| C(5)-Si(1) | 1.861(3) | C(9B)-H(9B) | 0.9500 |
| C(5)-H(5A) | 0.9800 | C(10B)-C(11B) | 1.409(4) |
| C(5)-H(5B) | 0.9800 | C(10B)-H(10B) | 0.9500 |
| C(5)-H(5C) | 0.9800 | C(11B)-H(11B) | 0.9500 |
| C(6)-Si(1) | 1.865(4) | C(17A)-C(18A) | 1.408(4) |
| C(6)-H(6A) | 0.9800 | C(17A)-C(21A) | 1.408(4) |
| C(6)-H(6B) | 0.9800 | C(17A)-H(17A) | 0.9500 |
| C(6)-H(6C) | 0.9800 | C(18A)-C(19A) | 1.408(4) |
| C(12)-C(16) | 1.390(5) | C(18A)-H(18A) | 0.9500 |
| C(12)-C(13) | 1.411(5) | C(19A)-C(20A) | 1.408(4) |
| C(12)-Ti(1) | 2.373(3) | C(19A)-H(19A) | 0.9500 |
| C(12)-H(12) | 0.9500 | C(20A)-C(21A) | 1.408(4) |
| C(13)-C(14) | 1.397(5) | C(20A)-H(20A) | 0.9500 |
| C(13)-Ti(1) | 2.397(3) | C(21A)-H(21A) | 0.9500 |
| C(13)-H(13) | 0.9500 | C(17B)-C(21B) | 1.391(6) |
| C(14)-C(15) | 1.400(5) | C(17B)-C(18B) | 1.391(6) |
| C(14)-Ti(1) | 2.416(3) | C(17B)-H(17B) | 0.9500 |
| C(14)-H(14) | 0.9500 | C(18B)-C(19B) | 1.391(6) |
| C(15)-C(16) | 1.411(5) | C(18B)-H(18B) | 0.9500 |
| C(15)-Ti(1) | 2.385(3) | C(19B)-C(20B) | 1.391(6) |
| C(15)-H(15) | 0.9500 | C(19B)-H(19B) | 0.9500 |
| C(16)-Ti(1) | 2.375(3) | C(20B)-C(21B) | 1.391(6) |
| C(16)-H(16) | 0.9500 | C(20B)-H(20B) | 0.9500 |
| C(22)-C(26) | 1.404(4) | C(21B)-H(21B) | 0.9500 |
| C(22)-C(23) | 1.417(5) | C(27A)-C(28A) | 1.3900 |
| C(22)-Zr(1) | 2.548(3) | C(27A)-C(32A) | 1.3900 |
| C(22)-H(22) | 0.9500 | C(27A)-C(33A) | 1.492(6) |
| C(23)-C(24) | 1.398(5) | C(28A)-C(29A) | 1.3900 |
| C(23)-Zr(1) | 2.552(3) | C(28A)-H(28A) | 0.9500 |
| C(23)-H(23) | 0.9500 | C(29A)-C(30A) | 1.3900 |
| C(24)-C(25) | 1.410(4) | C(29A)-H(29A) | 0.9500 |
| C(24)-Zr(1) | 2.545(3) | C(30A)-C(31A) | 1.3900 |
| C(24)-H(24) | 0.9500 | C(30A)-H(30A) | 0.9500 |
| C(25)-C(26) | 1.399(5) | C(31A)-C(32A) | 1.3900 |
| C(25)-Zr(1) | 2.572(3) | C(31A)-H(31A) | 0.9500 |
| C(25)-H(25) | 0.9500 | C(32A)-H(32A) | 0.9500 |
| C(26)-Zr(1) | 2.585(3) | C(33A)-H(33A) | 0.9800 |
| C(26)-H(26) | 0.9500 | C(33A)-H(33B) | 0.9800 |
| Ti(1)-C(8B) | 2.377(5) | C(33A)-H(33C) | 0.9800 |
| Ti(1)-C(7A) | 2.383(4) | C(27B)-C(28B) | 1.3900 |
| Ti(1)-C(7B) | 2.387(6) | C(27B)-C(32B) | 1.3900 |
| Ti(1)-C(9B) | 2.392(6) | C(27B)-C(33B) | 1.491(9) |
| Ti(1)-C(11A) | 2.392(5) | C(28B)-C(29B) | 1.3900 |
| Ti(1)-C(8A) | 2.403(4) | C(28B)-H(28B) | 0.9500 |
| Ti(1)-C(11B) | 2.408(7) | C(29B)-C(30B) | 1.3900 |
| Ti(1)-H(1A) | 2.03(4) | C(29B)-H(29B) | 0.9500 |
| Zr(1)-C(17B) | 2.508(9) | C(30B)-C(31B) | 1.3900 |
| Zr(1)-C(21B) | 2.522(8) | C(30B)-H(30B) | 0.9500 |
| Zr(1)-C(18B) | 2.537(8) | C(31B)-C(32B) | 1.3900 |
| Zr(1)-C(19A) | 2.555(4) | C(31B)-H(31B) | 0.9500 |
| Zr(1)-C(20B) | 2.559(8) | C(32B)-H(32B) | 0.9500 |
| Zr(1)-C(19B) | 2.568(7) | C(33B)-H(33D) | 0.9800 |
| C(7A)-C(11A) | 1.409(3) | C(33B)-H(33E) | 0.9800 |
| C(7A)-C(8A) | 1.409(3) | C(33B)-H(33F) | 0.9800 |
|  |  |  |  |
| Zr(1)-C(1)-Ti(1) | 85.61(9) | C(21B)-Zr(1)-C(23) | 120.7(2) |
| Zr(1)-C(1)-H(1A) | 137(2) | C(18B)-Zr(1)-C(23) | 81.5(2) |
| Ti(1)-C(1)-H(1A) | 51(2) | C(24)-Zr(1)-C(23) | 31.84(11) |
| Zr(1)-C(1)-H(1B) | 100(2) | C(22)-Zr(1)-C(23) | 32.26(11) |
| Ti(1)-C(1)-H(1B) | 121(2) | C(3)-Zr(1)-C(19A) | 103.24(19) |
| H(1A)-C(1)-H(1B) | 100(3) | C(2)-Zr(1)-C(19A) | 126.00(18) |
| Zr(1)-C(1)-H(1C) | 106(2) | C(1)-Zr(1)-C(19A) | 126.46(16) |
| Ti(1)-C(1)-H(1C) | 118(2) | C(24)-Zr(1)-C(19A) | 98.35(17) |
| H(1A)-C(1)-H(1C) | 99(3) | C(22)-Zr(1)-C(19A) | 83.54(13) |
| H(1B)-C(1)-H(1C) | 116(3) | C(23)-Zr(1)-C(19A) | 73.35(15) |
| C(3)-C(2)-Ti(1) | 171.8(2) | C(3)-Zr(1)-C(20B) | 129.0(2) |
| C(3)-C(2)-Zr(1) | 73.74(17) | C(2)-Zr(1)-C(20B) | 127.6(3) |
| Ti(1)-C(2)-Zr(1) | 98.34(10) | C(1)-Zr(1)-C(20B) | 82.6(2) |
| C(2)-C(3)-Si(1) | 140.0(2) | C(17B)-Zr(1)-C(20B) | 52.7(2) |
| C(2)-C(3)-Zr(1) | 74.88(17) | C(21B)-Zr(1)-C(20B) | 31.76(14) |
| Si(1)-C(3)-Zr(1) | 145.06(14) | C(18B)-Zr(1)-C(20B) | 52.4(2) |
| Si(1)-C(4)-H(4A) | 109.5 | C(24)-Zr(1)-C(20B) | 97.0(3) |
| Si(1)-C(4)-H(4B) | 109.5 | C(22)-Zr(1)-C(20B) | 117.9(3) |
| H(4A)-C(4)-H(4B) | 109.5 | C(23)-Zr(1)-C(20B) | 92.1(3) |
| Si(1)-C(4)-H(4C) | 109.5 | C(3)-Zr(1)-C(19B) | 121.4(3) |
| H(4A)-C(4)-H(4C) | 109.5 | C(2)-Zr(1)-C(19B) | 140.84(18) |
| H(4B)-C(4)-H(4C) | 109.5 | C(1)-Zr(1)-C(19B) | 110.7(3) |
| Si(1)-C(5)-H(5A) | 109.5 | C(17B)-Zr(1)-C(19B) | 52.6(2) |
| Si(1)-C(5)-H(5B) | 109.5 | C(21B)-Zr(1)-C(19B) | 52.5(2) |
| H(5A)-C(5)-H(5B) | 109.5 | C(18B)-Zr(1)-C(19B) | 31.61(14) |
| Si(1)-C(5)-H(5C) | 109.5 | C(24)-Zr(1)-C(19B) | 86.96(18) |
| H(5A)-C(5)-H(5C) | 109.5 | C(22)-Zr(1)-C(19B) | 88.1(3) |
| H(5B)-C(5)-H(5C) | 109.5 | C(23)-Zr(1)-C(19B) | 68.7(2) |
| Si(1)-C(6)-H(6A) | 109.5 | C(20B)-Zr(1)-C(19B) | 31.48(14) |
| Si(1)-C(6)-H(6B) | 109.5 | C(11A)-C(7A)-C(8A) | 108.0 |
| H(6A)-C(6)-H(6B) | 109.5 | C(11A)-C(7A)-Ti(1) | 73.17(17) |
| Si(1)-C(6)-H(6C) | 109.5 | C(8A)-C(7A)-Ti(1) | 73.63(17) |
| H(6A)-C(6)-H(6C) | 109.5 | C(11A)-C(7A)-H(7A) | 126.0 |
| H(6B)-C(6)-H(6C) | 109.5 | C(8A)-C(7A)-H(7A) | 126.0 |
| C(16)-C(12)-C(13) | 107.4(3) | Ti(1)-C(7A)-H(7A) | 119.1 |
| C(16)-C(12)-Ti(1) | 73.08(17) | C(9A)-C(8A)-C(7A) | 108.0 |
| C(13)-C(12)-Ti(1) | 73.75(16) | C(9A)-C(8A)-Ti(1) | 73.82(16) |
| C(16)-C(12)-H(12) | 126.3 | C(7A)-C(8A)-Ti(1) | 72.13(17) |
| C(13)-C(12)-H(12) | 126.3 | C(9A)-C(8A)-H(8A) | 126.0 |
| Ti(1)-C(12)-H(12) | 118.8 | C(7A)-C(8A)-H(8A) | 126.0 |
| C(14)-C(13)-C(12) | 108.2(3) | Ti(1)-C(8A)-H(8A) | 119.9 |
| C(14)-C(13)-Ti(1) | 73.88(17) | C(8A)-C(9A)-C(10A) | 108.0 |
| C(12)-C(13)-Ti(1) | 71.85(17) | C(8A)-C(9A)-Ti(1) | 72.23(17) |
| C(14)-C(13)-H(13) | 125.9 | C(10A)-C(9A)-Ti(1) | 72.82(17) |
| C(12)-C(13)-H(13) | 125.9 | C(8A)-C(9A)-H(9A) | 126.0 |
| Ti(1)-C(13)-H(13) | 120.2 | C(10A)-C(9A)-H(9A) | 126.0 |
| C(13)-C(14)-C(15) | 108.3(3) | Ti(1)-C(9A)-H(9A) | 120.7 |
| C(13)-C(14)-Ti(1) | 72.39(17) | C(11A)-C(10A)-C(9A) | 108.0 |
| C(15)-C(14)-Ti(1) | 71.82(17) | C(11A)-C(10A)-Ti(1) | 72.01(16) |
| C(13)-C(14)-H(14) | 125.9 | C(9A)-C(10A)-Ti(1) | 73.33(17) |
| C(15)-C(14)-H(14) | 125.9 | C(11A)-C(10A)-H(10A) | 126.0 |
| Ti(1)-C(14)-H(14) | 121.6 | C(9A)-C(10A)-H(10A) | 126.0 |
| C(14)-C(15)-C(16) | 107.3(3) | Ti(1)-C(10A)-H(10A) | 120.4 |
| C(14)-C(15)-Ti(1) | 74.28(17) | C(10A)-C(11A)-C(7A) | 108.0 |
| C(16)-C(15)-Ti(1) | 72.39(16) | C(10A)-C(11A)-Ti(1) | 73.92(16) |
| C(14)-C(15)-H(15) | 126.4 | C(7A)-C(11A)-Ti(1) | 72.51(17) |
| C(16)-C(15)-H(15) | 126.4 | C(10A)-C(11A)-H(11A) | 126.0 |
| Ti(1)-C(15)-H(15) | 118.9 | C(7A)-C(11A)-H(11A) | 126.0 |
| C(12)-C(16)-C(15) | 108.8(3) | Ti(1)-C(11A)-H(11A) | 119.4 |
| C(12)-C(16)-Ti(1) | 72.87(16) | C(11B)-C(7B)-C(8B) | 108.0 |
| C(15)-C(16)-Ti(1) | 73.12(17) | C(11B)-C(7B)-Ti(1) | 73.7(2) |
| C(12)-C(16)-H(16) | 125.6 | C(8B)-C(7B)-Ti(1) | 72.4(2) |
| C(15)-C(16)-H(16) | 125.6 | C(11B)-C(7B)-H(7B) | 126.0 |
| Ti(1)-C(16)-H(16) | 120.1 | C(8B)-C(7B)-H(7B) | 126.0 |
| C(26)-C(22)-C(23) | 107.6(3) | Ti(1)-C(7B)-H(7B) | 119.7 |
| C(26)-C(22)-Zr(1) | 75.58(17) | C(9B)-C(8B)-C(7B) | 108.0 |
| C(23)-C(22)-Zr(1) | 74.05(19) | C(9B)-C(8B)-Ti(1) | 73.4(2) |
| C(26)-C(22)-H(22) | 126.2 | C(7B)-C(8B)-Ti(1) | 73.2(2) |
| C(23)-C(22)-H(22) | 126.2 | C(9B)-C(8B)-H(8B) | 126.0 |
| Zr(1)-C(22)-H(22) | 116.3 | C(7B)-C(8B)-H(8B) | 126.0 |
| C(24)-C(23)-C(22) | 108.2(3) | Ti(1)-C(8B)-H(8B) | 119.3 |
| C(24)-C(23)-Zr(1) | 73.77(18) | C(10B)-C(9B)-C(8B) | 108.0 |
| C(22)-C(23)-Zr(1) | 73.69(17) | C(10B)-C(9B)-Ti(1) | 73.7(2) |
| C(24)-C(23)-H(23) | 125.9 | C(8B)-C(9B)-Ti(1) | 72.2(2) |
| C(22)-C(23)-H(23) | 125.9 | C(10B)-C(9B)-H(9B) | 126.0 |
| Zr(1)-C(23)-H(23) | 118.5 | C(8B)-C(9B)-H(9B) | 126.0 |
| C(23)-C(24)-C(25) | 107.6(3) | Ti(1)-C(9B)-H(9B) | 119.9 |
| C(23)-C(24)-Zr(1) | 74.38(18) | C(11B)-C(10B)-C(9B) | 108.0 |
| C(25)-C(24)-Zr(1) | 75.07(17) | C(11B)-C(10B)-Ti(1) | 72.9(2) |
| C(23)-C(24)-H(24) | 126.2 | C(9B)-C(10B)-Ti(1) | 72.2(2) |
| C(25)-C(24)-H(24) | 126.2 | C(11B)-C(10B)-H(10B) | 126.0 |
| Zr(1)-C(24)-H(24) | 116.5 | C(9B)-C(10B)-H(10B) | 126.0 |
| C(26)-C(25)-C(24) | 108.5(3) | Ti(1)-C(10B)-H(10B) | 120.7 |
| C(26)-C(25)-Zr(1) | 74.77(17) | C(10B)-C(11B)-C(7B) | 108.0 |
| C(24)-C(25)-Zr(1) | 72.95(18) | C(10B)-C(11B)-Ti(1) | 73.1(2) |
| C(26)-C(25)-H(25) | 125.8 | C(7B)-C(11B)-Ti(1) | 72.1(2) |
| C(24)-C(25)-H(25) | 125.8 | C(10B)-C(11B)-H(11B) | 126.0 |
| Zr(1)-C(25)-H(25) | 118.4 | C(7B)-C(11B)-H(11B) | 126.0 |
| C(25)-C(26)-C(22) | 108.1(3) | Ti(1)-C(11B)-H(11B) | 120.6 |
| C(25)-C(26)-Zr(1) | 73.76(17) | C(18A)-C(17A)-C(21A) | 108.0 |
| C(22)-C(26)-Zr(1) | 72.69(18) | C(18A)-C(17A)-Zr(1) | 73.02(16) |
| C(25)-C(26)-H(26) | 126.0 | C(21A)-C(17A)-Zr(1) | 74.43(16) |
| C(22)-C(26)-H(26) | 126.0 | C(18A)-C(17A)-H(17A) | 126.0 |
| Zr(1)-C(26)-H(26) | 119.4 | C(21A)-C(17A)-H(17A) | 126.0 |
| C(3)-Si(1)-C(5) | 111.45(14) | Zr(1)-C(17A)-H(17A) | 118.5 |
| C(3)-Si(1)-C(6) | 110.50(15) | C(19A)-C(18A)-C(17A) | 108.0 |
| C(5)-Si(1)-C(6) | 108.41(18) | C(19A)-C(18A)-Zr(1) | 73.45(16) |
| C(3)-Si(1)-C(4) | 108.57(16) | C(17A)-C(18A)-Zr(1) | 75.38(16) |
| C(5)-Si(1)-C(4) | 108.9(2) | C(19A)-C(18A)-H(18A) | 126.0 |
| C(6)-Si(1)-C(4) | 108.9(2) | C(17A)-C(18A)-H(18A) | 126.0 |
| C(2)-Ti(1)-C(12) | 81.81(10) | Zr(1)-C(18A)-H(18A) | 117.2 |
| C(2)-Ti(1)-C(16) | 113.85(11) | C(18A)-C(19A)-C(20A) | 108.0 |
| C(12)-Ti(1)-C(16) | 34.04(11) | C(18A)-C(19A)-Zr(1) | 74.66(16) |
| C(2)-Ti(1)-C(8B) | 118.1(2) | C(20A)-C(19A)-Zr(1) | 74.88(16) |
| C(12)-Ti(1)-C(8B) | 97.09(17) | C(18A)-C(19A)-H(19A) | 126.0 |
| C(16)-Ti(1)-C(8B) | 72.30(15) | C(20A)-C(19A)-H(19A) | 126.0 |
| C(2)-Ti(1)-C(7A) | 130.47(13) | Zr(1)-C(19A)-H(19A) | 116.5 |
| C(12)-Ti(1)-C(7A) | 137.35(15) | C(21A)-C(20A)-C(19A) | 108.0 |
| C(16)-Ti(1)-C(7A) | 104.20(15) | C(21A)-C(20A)-Zr(1) | 75.33(16) |
| C(2)-Ti(1)-C(15) | 135.71(11) | C(19A)-C(20A)-Zr(1) | 73.26(17) |
| C(12)-Ti(1)-C(15) | 57.20(11) | C(21A)-C(20A)-H(20A) | 126.0 |
| C(16)-Ti(1)-C(15) | 34.49(12) | C(19A)-C(20A)-H(20A) | 126.0 |
| C(8B)-Ti(1)-C(15) | 85.60(18) | Zr(1)-C(20A)-H(20A) | 117.4 |
| C(7A)-Ti(1)-C(15) | 93.36(13) | C(20A)-C(21A)-C(17A) | 108.0 |
| C(2)-Ti(1)-C(7B) | 133.40(16) | C(20A)-C(21A)-Zr(1) | 73.13(16) |
| C(12)-Ti(1)-C(7B) | 125.5(2) | C(17A)-C(21A)-Zr(1) | 74.18(16) |
| C(16)-Ti(1)-C(7B) | 93.39(19) | C(20A)-C(21A)-H(21A) | 126.0 |
| C(8B)-Ti(1)-C(7B) | 34.40(11) | C(17A)-C(21A)-H(21A) | 126.0 |
| C(15)-Ti(1)-C(7B) | 87.86(16) | Zr(1)-C(21A)-H(21A) | 118.6 |
| C(2)-Ti(1)-C(9B) | 84.3(2) | C(21B)-C(17B)-C(18B) | 108.0 |
| C(12)-Ti(1)-C(9B) | 99.20(16) | C(21B)-C(17B)-Zr(1) | 74.5(3) |
| C(16)-Ti(1)-C(9B) | 90.82(19) | C(18B)-C(17B)-Zr(1) | 75.1(3) |
| C(8B)-Ti(1)-C(9B) | 34.37(11) | C(21B)-C(17B)-H(17B) | 126.0 |
| C(15)-Ti(1)-C(9B) | 115.7(2) | C(18B)-C(17B)-H(17B) | 126.0 |
| C(7B)-Ti(1)-C(9B) | 56.98(17) | Zr(1)-C(17B)-H(17B) | 116.4 |
| C(2)-Ti(1)-C(11A) | 96.47(13) | C(17B)-C(18B)-C(19B) | 108.0 |
| C(12)-Ti(1)-C(11A) | 153.97(13) | C(17B)-C(18B)-Zr(1) | 72.9(3) |
| C(16)-Ti(1)-C(11A) | 132.14(13) | C(19B)-C(18B)-Zr(1) | 75.4(3) |
| C(7A)-Ti(1)-C(11A) | 34.32(8) | C(17B)-C(18B)-H(18B) | 126.0 |
| C(15)-Ti(1)-C(11A) | 127.65(14) | C(19B)-C(18B)-H(18B) | 126.0 |
| C(2)-Ti(1)-C(13) | 80.42(10) | Zr(1)-C(18B)-H(18B) | 117.7 |
| C(12)-Ti(1)-C(13) | 34.41(11) | C(20B)-C(19B)-C(18B) | 108.0 |
| C(16)-Ti(1)-C(13) | 56.46(11) | C(20B)-C(19B)-Zr(1) | 73.9(3) |
| C(8B)-Ti(1)-C(13) | 128.30(15) | C(18B)-C(19B)-Zr(1) | 72.9(3) |
| C(7A)-Ti(1)-C(13) | 148.95(13) | C(20B)-C(19B)-H(19B) | 126.0 |
| C(15)-Ti(1)-C(13) | 56.59(11) | C(18B)-C(19B)-H(19B) | 126.0 |
| C(7B)-Ti(1)-C(13) | 144.34(16) | Zr(1)-C(19B)-H(19B) | 119.0 |
| C(9B)-Ti(1)-C(13) | 132.62(15) | C(19B)-C(20B)-C(21B) | 108.0 |
| C(11A)-Ti(1)-C(13) | 170.92(12) | C(19B)-C(20B)-Zr(1) | 74.6(3) |
| C(2)-Ti(1)-C(8A) | 130.91(16) | C(21B)-C(20B)-Zr(1) | 72.6(3) |
| C(12)-Ti(1)-C(8A) | 105.14(14) | C(19B)-C(20B)-H(20B) | 126.0 |
| C(16)-Ti(1)-C(8A) | 75.50(13) | C(21B)-C(20B)-H(20B) | 126.0 |
| C(7A)-Ti(1)-C(8A) | 34.24(8) | Zr(1)-C(20B)-H(20B) | 118.7 |
| C(15)-Ti(1)-C(8A) | 80.15(13) | C(17B)-C(21B)-C(20B) | 108.0 |
| C(11A)-Ti(1)-C(8A) | 56.77(13) | C(17B)-C(21B)-Zr(1) | 73.4(3) |
| C(13)-Ti(1)-C(8A) | 131.36(12) | C(20B)-C(21B)-Zr(1) | 75.6(3) |
| C(2)-Ti(1)-C(11B) | 104.54(17) | C(17B)-C(21B)-H(21B) | 126.0 |
| C(12)-Ti(1)-C(11B) | 153.39(16) | C(20B)-C(21B)-H(21B) | 126.0 |
| C(16)-Ti(1)-C(11B) | 126.48(17) | Zr(1)-C(21B)-H(21B) | 117.1 |
| C(8B)-Ti(1)-C(11B) | 56.89(17) | C(28A)-C(27A)-C(32A) | 120.0 |
| C(15)-Ti(1)-C(11B) | 119.58(17) | C(28A)-C(27A)-C(33A) | 118.2(4) |
| C(7B)-Ti(1)-C(11B) | 34.16(11) | C(32A)-C(27A)-C(33A) | 121.8(4) |
| C(9B)-Ti(1)-C(11B) | 56.71(17) | C(27A)-C(28A)-C(29A) | 120.0 |
| C(13)-Ti(1)-C(11B) | 170.39(16) | C(27A)-C(28A)-H(28A) | 120.0 |
| C(2)-Ti(1)-H(1A) | 112.5(11) | C(29A)-C(28A)-H(28A) | 120.0 |
| C(12)-Ti(1)-H(1A) | 126.5(11) | C(30A)-C(29A)-C(28A) | 120.0 |
| C(16)-Ti(1)-H(1A) | 118.2(11) | C(30A)-C(29A)-H(29A) | 120.0 |
| C(8B)-Ti(1)-H(1A) | 116.4(11) | C(28A)-C(29A)-H(29A) | 120.0 |
| C(7A)-Ti(1)-H(1A) | 72.0(11) | C(31A)-C(30A)-C(29A) | 120.0 |
| C(15)-Ti(1)-H(1A) | 83.8(11) | C(31A)-C(30A)-H(30A) | 120.0 |
| C(7B)-Ti(1)-H(1A) | 82.6(11) | C(29A)-C(30A)-H(30A) | 120.0 |
| C(9B)-Ti(1)-H(1A) | 132.2(11) | C(30A)-C(31A)-C(32A) | 120.0 |
| C(11A)-Ti(1)-H(1A) | 78.3(11) | C(30A)-C(31A)-H(31A) | 120.0 |
| C(13)-Ti(1)-H(1A) | 94.9(11) | C(32A)-C(31A)-H(31A) | 120.0 |
| C(8A)-Ti(1)-H(1A) | 101.9(11) | C(31A)-C(32A)-C(27A) | 120.0 |
| C(11B)-Ti(1)-H(1A) | 75.6(11) | C(31A)-C(32A)-H(32A) | 120.0 |
| C(3)-Zr(1)-C(2) | 31.38(9) | C(27A)-C(32A)-H(32A) | 120.0 |
| C(3)-Zr(1)-C(1) | 116.93(10) | C(27A)-C(33A)-H(33A) | 109.5 |
| C(2)-Zr(1)-C(1) | 85.59(9) | C(27A)-C(33A)-H(33B) | 109.5 |
| C(3)-Zr(1)-C(17B) | 76.9(2) | H(33A)-C(33A)-H(33B) | 109.5 |
| C(2)-Zr(1)-C(17B) | 88.27(19) | C(27A)-C(33A)-H(33C) | 109.5 |
| C(1)-Zr(1)-C(17B) | 115.0(3) | H(33A)-C(33A)-H(33C) | 109.5 |
| C(3)-Zr(1)-C(21B) | 99.9(3) | H(33B)-C(33A)-H(33C) | 109.5 |
| C(2)-Zr(1)-C(21B) | 96.5(3) | C(28B)-C(27B)-C(32B) | 120.0 |
| C(1)-Zr(1)-C(21B) | 84.7(2) | C(28B)-C(27B)-C(33B) | 124.4(11) |
| C(17B)-Zr(1)-C(21B) | 32.10(15) | C(32B)-C(27B)-C(33B) | 115.5(11) |
| C(3)-Zr(1)-C(18B) | 89.8(3) | C(27B)-C(28B)-C(29B) | 120.0 |
| C(2)-Zr(1)-C(18B) | 112.7(3) | C(27B)-C(28B)-H(28B) | 120.0 |
| C(1)-Zr(1)-C(18B) | 134.08(19) | C(29B)-C(28B)-H(28B) | 120.0 |
| C(17B)-Zr(1)-C(18B) | 32.00(15) | C(28B)-C(29B)-C(30B) | 120.0 |
| C(21B)-Zr(1)-C(18B) | 52.8(2) | C(28B)-C(29B)-H(29B) | 120.0 |
| C(3)-Zr(1)-C(24) | 130.43(10) | C(30B)-C(29B)-H(29B) | 120.0 |
| C(2)-Zr(1)-C(24) | 131.50(10) | C(31B)-C(30B)-C(29B) | 120.0 |
| C(1)-Zr(1)-C(24) | 81.97(11) | C(31B)-C(30B)-H(30B) | 120.0 |
| C(17B)-Zr(1)-C(24) | 139.1(2) | C(29B)-C(30B)-H(30B) | 120.0 |
| C(21B)-Zr(1)-C(24) | 128.4(3) | C(30B)-C(31B)-C(32B) | 120.0 |
| C(18B)-Zr(1)-C(24) | 109.4(3) | C(30B)-C(31B)-H(31B) | 120.0 |
| C(3)-Zr(1)-C(22) | 85.50(10) | C(32B)-C(31B)-H(31B) | 120.0 |
| C(2)-Zr(1)-C(22) | 108.22(10) | C(31B)-C(32B)-C(27B) | 120.0 |
| C(1)-Zr(1)-C(22) | 130.93(11) | C(31B)-C(32B)-H(32B) | 120.0 |
| C(17B)-Zr(1)-C(22) | 112.4(3) | C(27B)-C(32B)-H(32B) | 120.0 |
| C(21B)-Zr(1)-C(22) | 136.8(2) | C(27B)-C(33B)-H(33D) | 109.5 |
| C(18B)-Zr(1)-C(22) | 84.7(2) | C(27B)-C(33B)-H(33E) | 109.5 |
| C(24)-Zr(1)-C(22) | 53.20(11) | H(33D)-C(33B)-H(33E) | 109.5 |
| C(3)-Zr(1)-C(23) | 117.49(10) | C(27B)-C(33B)-H(33F) | 109.5 |
| C(2)-Zr(1)-C(23) | 139.02(10) | H(33D)-C(33B)-H(33F) | 109.5 |
| C(1)-Zr(1)-C(23) | 112.57(11) | H(33E)-C(33B)-H(33F) | 109.5 |
| C(17B)-Zr(1)-C(23) | 113.5(2) |  |  |

## 2.2 Crystallographic details of complex 8

Hydrogen atoms attached to C1 could be found from the difference Fourier map and were refined freely.

**Figure S2.** Labeling scheme of the molecular structure of **8**. Displacement ellipsoids correspond to 30% probability. Hydrogen atoms are omitted for clarity. Lower occupied parts of disorder are shown with unfilled lines.

**Table S3.** Bond lengths [Å] and angles [°] for complex **8**.

| C(1)-Zr(1) | 2.429(3) | C(16)-Zr(1) | 2.557(3) |
| --- | --- | --- | --- |
| C(1)-Ti(1) | 2.496(3) | C(16)-H(16) | 0.9500 |
| C(1)-H(1A) | 0.89(4) | C(17)-C(18) | 1.405(6) |
| C(1)-H(1B) | 0.96(5) | C(17)-Zr(1) | 2.578(4) |
| C(1)-H(1C) | 0.98(5) | C(17)-H(17) | 0.9500 |
| C(2)-C(3) | 1.281(5) | C(18)-C(19) | 1.394(6) |
| C(2)-Ti(1) | 2.005(3) | C(18)-Zr(1) | 2.548(4) |
| C(2)-Zr(1) | 2.349(3) | C(18)-H(18) | 0.9500 |
| C(3)-C(4) | 1.460(4) | C(19)-Zr(1) | 2.561(3) |
| C(3)-Zr(1) | 2.292(3) | C(19)-H(19) | 0.9500 |
| C(4)-C(9) | 1.398(5) | C(20)-C(21) | 1.395(6) |
| C(4)-C(5) | 1.404(4) | C(20)-C(24) | 1.403(5) |
| C(5)-C(6) | 1.381(5) | C(20)-Ti(1) | 2.404(3) |
| C(5)-H(5) | 0.9500 | C(20)-H(20) | 0.9500 |
| C(6)-C(7) | 1.389(5) | C(21)-C(22) | 1.418(5) |
| C(6)-H(6) | 0.9500 | C(21)-Ti(1) | 2.391(3) |
| C(7)-C(8) | 1.386(5) | C(21)-H(21) | 0.9500 |
| C(7)-H(7) | 0.9500 | C(22)-C(23) | 1.399(6) |
| C(8)-C(9) | 1.386(5) | C(22)-Ti(1) | 2.386(4) |
| C(8)-H(8) | 0.9500 | C(22)-H(22) | 0.9500 |
| C(9)-H(9) | 0.9500 | C(23)-C(24) | 1.404(6) |
| C(10)-C(11) | 1.404(5) | C(23)-Ti(1) | 2.414(4) |
| C(10)-C(14) | 1.404(6) | C(23)-H(23) | 0.9500 |
| C(10)-Zr(1) | 2.582(3) | C(24)-Ti(1) | 2.398(3) |
| C(10)-H(10) | 0.9500 | C(24)-H(24) | 0.9500 |
| C(11)-C(12) | 1.410(5) | C(25)-C(29) | 1.400(6) |
| C(11)-Zr(1) | 2.555(3) | C(25)-C(26) | 1.406(5) |
| C(11)-H(11) | 0.9500 | C(25)-Ti(1) | 2.403(3) |
| C(12)-C(13) | 1.413(5) | C(25)-H(25) | 0.9500 |
| C(12)-Zr(1) | 2.542(3) | C(26)-C(27) | 1.408(5) |
| C(12)-H(12) | 0.9500 | C(26)-Ti(1) | 2.379(3) |
| C(13)-C(14) | 1.396(5) | C(26)-H(26) | 0.9500 |
| C(13)-Zr(1) | 2.547(3) | C(27)-C(28) | 1.412(5) |
| C(13)-H(13) | 0.9500 | C(27)-Ti(1) | 2.387(3) |
| C(14)-Zr(1) | 2.575(3) | C(27)-H(27) | 0.9500 |
| C(14)-H(14) | 0.9500 | C(28)-C(29) | 1.398(6) |
| C(15)-C(19) | 1.400(5) | C(28)-Ti(1) | 2.428(4) |
| C(15)-C(16) | 1.414(5) | C(28)-H(28) | 0.9500 |
| C(15)-Zr(1) | 2.535(3) | C(29)-Ti(1) | 2.447(3) |
| C(15)-H(15) | 0.9500 | C(29)-H(29) | 0.9500 |
| C(16)-C(17) | 1.393(6) | Ti(1)-H(1A) | 2.05(4) |
|  |  |  |  |
| Zr(1)-C(1)-Ti(1) | 85.08(10) | C(28)-C(29)-Ti(1) | 72.59(19) |
| Zr(1)-C(1)-H(1A) | 136(3) | C(25)-C(29)-Ti(1) | 71.47(19) |
| Ti(1)-C(1)-H(1A) | 51(3) | C(28)-C(29)-H(29) | 125.8 |
| Zr(1)-C(1)-H(1B) | 101(3) | C(25)-C(29)-H(29) | 125.8 |
| Ti(1)-C(1)-H(1B) | 127(3) | Ti(1)-C(29)-H(29) | 121.9 |
| H(1A)-C(1)-H(1B) | 105(4) | C(2)-Ti(1)-C(26) | 117.62(12) |
| Zr(1)-C(1)-H(1C) | 106(3) | C(2)-Ti(1)-C(22) | 129.64(13) |
| Ti(1)-C(1)-H(1C) | 122(3) | C(26)-Ti(1)-C(22) | 100.26(14) |
| H(1A)-C(1)-H(1C) | 100(4) | C(2)-Ti(1)-C(27) | 84.01(12) |
| H(1B)-C(1)-H(1C) | 107(4) | C(26)-Ti(1)-C(27) | 34.37(13) |
| C(3)-C(2)-Ti(1) | 171.0(2) | C(22)-Ti(1)-C(27) | 131.71(14) |
| C(3)-C(2)-Zr(1) | 71.53(18) | C(2)-Ti(1)-C(21) | 96.01(12) |
| Ti(1)-C(2)-Zr(1) | 99.47(12) | C(26)-Ti(1)-C(21) | 130.75(13) |
| C(2)-C(3)-C(4) | 138.9(3) | C(22)-Ti(1)-C(21) | 34.54(13) |
| C(2)-C(3)-Zr(1) | 76.46(19) | C(27)-Ti(1)-C(21) | 150.33(13) |
| C(4)-C(3)-Zr(1) | 144.6(2) | C(2)-Ti(1)-C(24) | 93.14(13) |
| C(9)-C(4)-C(5) | 118.4(3) | C(26)-Ti(1)-C(24) | 85.16(13) |
| C(9)-C(4)-C(3) | 122.2(3) | C(22)-Ti(1)-C(24) | 56.55(14) |
| C(5)-C(4)-C(3) | 119.4(3) | C(27)-Ti(1)-C(24) | 93.90(14) |
| C(6)-C(5)-C(4) | 120.7(3) | C(21)-Ti(1)-C(24) | 56.44(13) |
| C(6)-C(5)-H(5) | 119.6 | C(2)-Ti(1)-C(25) | 133.94(13) |
| C(4)-C(5)-H(5) | 119.6 | C(26)-Ti(1)-C(25) | 34.20(12) |
| C(5)-C(6)-C(7) | 120.3(3) | C(22)-Ti(1)-C(25) | 96.08(14) |
| C(5)-C(6)-H(6) | 119.9 | C(27)-Ti(1)-C(25) | 56.88(13) |
| C(7)-C(6)-H(6) | 119.9 | C(21)-Ti(1)-C(25) | 130.05(13) |
| C(8)-C(7)-C(6) | 119.6(3) | C(24)-Ti(1)-C(25) | 111.21(12) |
| C(8)-C(7)-H(7) | 120.2 | C(2)-Ti(1)-C(20) | 75.61(12) |
| C(6)-C(7)-H(7) | 120.2 | C(26)-Ti(1)-C(20) | 119.02(12) |
| C(9)-C(8)-C(7) | 120.4(3) | C(22)-Ti(1)-C(20) | 56.70(13) |
| C(9)-C(8)-H(8) | 119.8 | C(27)-Ti(1)-C(20) | 119.88(13) |
| C(7)-C(8)-H(8) | 119.8 | C(21)-Ti(1)-C(20) | 33.82(14) |
| C(8)-C(9)-C(4) | 120.6(3) | C(24)-Ti(1)-C(20) | 33.97(12) |
| C(8)-C(9)-H(9) | 119.7 | C(25)-Ti(1)-C(20) | 142.63(12) |
| C(4)-C(9)-H(9) | 119.7 | C(2)-Ti(1)-C(23) | 126.80(13) |
| C(11)-C(10)-C(14) | 108.0(3) | C(26)-Ti(1)-C(23) | 74.32(13) |
| C(11)-C(10)-Zr(1) | 73.09(19) | C(22)-Ti(1)-C(23) | 33.88(14) |
| C(14)-C(10)-Zr(1) | 73.9(2) | C(27)-Ti(1)-C(23) | 100.01(14) |
| C(11)-C(10)-H(10) | 126.0 | C(21)-Ti(1)-C(23) | 56.53(13) |
| C(14)-C(10)-H(10) | 126.0 | C(24)-Ti(1)-C(23) | 33.91(14) |
| Zr(1)-C(10)-H(10) | 118.9 | C(25)-Ti(1)-C(23) | 86.62(13) |
| C(10)-C(11)-C(12) | 107.9(3) | C(20)-Ti(1)-C(23) | 56.33(13) |
| C(10)-C(11)-Zr(1) | 75.20(19) | C(2)-Ti(1)-C(28) | 77.99(12) |
| C(12)-C(11)-Zr(1) | 73.43(18) | C(26)-Ti(1)-C(28) | 56.39(12) |
| C(10)-C(11)-H(11) | 126.1 | C(22)-Ti(1)-C(28) | 152.07(13) |
| C(12)-C(11)-H(11) | 126.1 | C(27)-Ti(1)-C(28) | 34.08(13) |
| Zr(1)-C(11)-H(11) | 117.3 | C(21)-Ti(1)-C(28) | 172.73(12) |
| C(11)-C(12)-C(13) | 107.8(3) | C(24)-Ti(1)-C(28) | 127.38(13) |
| C(11)-C(12)-Zr(1) | 74.45(18) | C(25)-Ti(1)-C(28) | 56.06(13) |
| C(13)-C(12)-Zr(1) | 74.07(18) | C(20)-Ti(1)-C(28) | 145.10(14) |
| C(11)-C(12)-H(12) | 126.1 | C(23)-Ti(1)-C(28) | 130.46(13) |
| C(13)-C(12)-H(12) | 126.1 | C(2)-Ti(1)-C(29) | 106.04(14) |
| Zr(1)-C(12)-H(12) | 117.4 | C(26)-Ti(1)-C(29) | 56.01(11) |
| C(14)-C(13)-C(12) | 107.8(3) | C(22)-Ti(1)-C(29) | 122.89(15) |
| C(14)-C(13)-Zr(1) | 75.26(19) | C(27)-Ti(1)-C(29) | 56.13(13) |
| C(12)-C(13)-Zr(1) | 73.70(18) | C(21)-Ti(1)-C(29) | 148.78(14) |
| C(14)-C(13)-H(13) | 126.1 | C(24)-Ti(1)-C(29) | 141.10(12) |
| C(12)-C(13)-H(13) | 126.1 | C(25)-Ti(1)-C(29) | 33.54(13) |
| Zr(1)-C(13)-H(13) | 117.0 | C(20)-Ti(1)-C(29) | 175.02(11) |
| C(13)-C(14)-C(10) | 108.5(3) | C(23)-Ti(1)-C(29) | 120.15(13) |
| C(13)-C(14)-Zr(1) | 73.1(2) | C(28)-Ti(1)-C(29) | 33.32(15) |
| C(10)-C(14)-Zr(1) | 74.50(19) | C(2)-Ti(1)-C(1) | 90.70(11) |
| C(13)-C(14)-H(14) | 125.7 | C(26)-Ti(1)-C(1) | 130.57(11) |
| C(10)-C(14)-H(14) | 125.7 | C(22)-Ti(1)-C(1) | 88.20(12) |
| Zr(1)-C(14)-H(14) | 118.5 | C(27)-Ti(1)-C(1) | 130.17(12) |
| C(19)-C(15)-C(16) | 107.1(3) | C(21)-Ti(1)-C(1) | 79.48(12) |
| C(19)-C(15)-Zr(1) | 75.08(19) | C(24)-Ti(1)-C(1) | 135.92(12) |
| C(16)-C(15)-Zr(1) | 74.74(19) | C(25)-Ti(1)-C(1) | 96.80(11) |
| C(19)-C(15)-H(15) | 126.5 | C(20)-Ti(1)-C(1) | 106.34(12) |
| C(16)-C(15)-H(15) | 126.5 | C(23)-Ti(1)-C(1) | 121.64(14) |
| Zr(1)-C(15)-H(15) | 116.0 | C(28)-Ti(1)-C(1) | 96.35(12) |
| C(17)-C(16)-C(15) | 108.0(3) | C(29)-Ti(1)-C(1) | 78.44(10) |
| C(17)-C(16)-Zr(1) | 75.1(2) | C(2)-Ti(1)-H(1A) | 110.2(13) |
| C(15)-C(16)-Zr(1) | 73.01(19) | C(26)-Ti(1)-H(1A) | 116.4(12) |
| C(17)-C(16)-H(16) | 126.0 | C(22)-Ti(1)-H(1A) | 76.7(12) |
| C(15)-C(16)-H(16) | 126.0 | C(27)-Ti(1)-H(1A) | 128.8(12) |
| Zr(1)-C(16)-H(16) | 117.9 | C(21)-Ti(1)-H(1A) | 79.1(12) |
| C(16)-C(17)-C(18) | 108.4(3) | C(24)-Ti(1)-H(1A) | 131.9(12) |
| C(16)-C(17)-Zr(1) | 73.5(2) | C(25)-Ti(1)-H(1A) | 82.3(12) |
| C(18)-C(17)-Zr(1) | 72.9(2) | C(20)-Ti(1)-H(1A) | 111.3(12) |
| C(16)-C(17)-H(17) | 125.8 | C(23)-Ti(1)-H(1A) | 107.6(12) |
| C(18)-C(17)-H(17) | 125.8 | C(28)-Ti(1)-H(1A) | 99.0(12) |
| Zr(1)-C(17)-H(17) | 119.6 | C(29)-Ti(1)-H(1A) | 72.7(12) |
| C(19)-C(18)-C(17) | 107.5(3) | C(1)-Ti(1)-H(1A) | 19.6(12) |
| C(19)-C(18)-Zr(1) | 74.7(2) | C(3)-Zr(1)-C(2) | 32.01(11) |
| C(17)-C(18)-Zr(1) | 75.3(2) | C(3)-Zr(1)-C(1) | 116.72(11) |
| C(19)-C(18)-H(18) | 126.3 | C(2)-Zr(1)-C(1) | 84.73(10) |
| C(17)-C(18)-H(18) | 126.3 | C(3)-Zr(1)-C(15) | 129.08(12) |
| Zr(1)-C(18)-H(18) | 116.0 | C(2)-Zr(1)-C(15) | 128.05(11) |
| C(18)-C(19)-C(15) | 108.9(3) | C(1)-Zr(1)-C(15) | 80.04(11) |
| C(18)-C(19)-Zr(1) | 73.6(2) | C(3)-Zr(1)-C(12) | 103.60(11) |
| C(15)-C(19)-Zr(1) | 73.04(19) | C(2)-Zr(1)-C(12) | 127.62(11) |
| C(18)-C(19)-H(19) | 125.5 | C(1)-Zr(1)-C(12) | 127.41(10) |
| C(15)-C(19)-H(19) | 125.5 | C(15)-Zr(1)-C(12) | 100.25(11) |
| Zr(1)-C(19)-H(19) | 119.6 | C(3)-Zr(1)-C(13) | 130.78(11) |
| C(21)-C(20)-C(24) | 108.1(3) | C(2)-Zr(1)-C(13) | 140.61(11) |
| C(21)-C(20)-Ti(1) | 72.6(2) | C(1)-Zr(1)-C(13) | 95.63(11) |
| C(24)-C(20)-Ti(1) | 72.8(2) | C(15)-Zr(1)-C(13) | 90.42(12) |
| C(21)-C(20)-H(20) | 126.0 | C(12)-Zr(1)-C(13) | 32.23(11) |
| C(24)-C(20)-H(20) | 126.0 | C(3)-Zr(1)-C(18) | 88.60(13) |
| Ti(1)-C(20)-H(20) | 120.5 | C(2)-Zr(1)-C(18) | 112.31(12) |
| C(20)-C(21)-C(22) | 107.9(3) | C(1)-Zr(1)-C(18) | 131.38(11) |
| C(20)-C(21)-Ti(1) | 73.6(2) | C(15)-Zr(1)-C(18) | 53.14(12) |
| C(22)-C(21)-Ti(1) | 72.6(2) | C(12)-Zr(1)-C(18) | 78.72(12) |
| C(20)-C(21)-H(21) | 126.0 | C(13)-Zr(1)-C(18) | 96.88(13) |
| C(22)-C(21)-H(21) | 126.0 | C(3)-Zr(1)-C(11) | 77.66(11) |
| Ti(1)-C(21)-H(21) | 119.6 | C(2)-Zr(1)-C(11) | 96.14(11) |
| C(23)-C(22)-C(21) | 107.7(3) | C(1)-Zr(1)-C(11) | 127.62(11) |
| C(23)-C(22)-Ti(1) | 74.2(2) | C(15)-Zr(1)-C(11) | 131.94(12) |
| C(21)-C(22)-Ti(1) | 72.9(2) | C(12)-Zr(1)-C(11) | 32.12(10) |
| C(23)-C(22)-H(22) | 126.1 | C(13)-Zr(1)-C(11) | 53.12(11) |
| C(21)-C(22)-H(22) | 126.1 | C(18)-Zr(1)-C(11) | 96.69(12) |
| Ti(1)-C(22)-H(22) | 118.7 | C(3)-Zr(1)-C(16) | 99.47(11) |
| C(22)-C(23)-C(24) | 108.0(3) | C(2)-Zr(1)-C(16) | 96.54(11) |
| C(22)-C(23)-Ti(1) | 72.0(2) | C(1)-Zr(1)-C(16) | 81.20(11) |
| C(24)-C(23)-Ti(1) | 72.4(2) | C(15)-Zr(1)-C(16) | 32.25(12) |
| C(22)-C(23)-H(23) | 126.0 | C(12)-Zr(1)-C(16) | 125.19(11) |
| C(24)-C(23)-H(23) | 126.0 | C(13)-Zr(1)-C(16) | 122.53(12) |
| Ti(1)-C(23)-H(23) | 121.3 | C(18)-Zr(1)-C(16) | 52.78(12) |
| C(20)-C(24)-C(23) | 108.3(4) | C(11)-Zr(1)-C(16) | 149.46(12) |
| C(20)-C(24)-Ti(1) | 73.23(19) | C(3)-Zr(1)-C(19) | 120.27(13) |
| C(23)-C(24)-Ti(1) | 73.7(2) | C(2)-Zr(1)-C(19) | 140.64(11) |
| C(20)-C(24)-H(24) | 125.9 | C(1)-Zr(1)-C(19) | 109.58(12) |
| C(23)-C(24)-H(24) | 125.9 | C(15)-Zr(1)-C(19) | 31.88(12) |
| Ti(1)-C(24)-H(24) | 119.1 | C(12)-Zr(1)-C(19) | 72.99(11) |
| C(29)-C(25)-C(26) | 107.8(3) | C(13)-Zr(1)-C(19) | 76.14(12) |
| C(29)-C(25)-Ti(1) | 74.99(18) | C(18)-Zr(1)-C(19) | 31.67(14) |
| C(26)-C(25)-Ti(1) | 71.98(19) | C(11)-Zr(1)-C(19) | 102.28(12) |
| C(29)-C(25)-H(25) | 126.1 | C(16)-Zr(1)-C(19) | 52.49(12) |
| C(26)-C(25)-H(25) | 126.1 | C(3)-Zr(1)-C(14) | 117.27(12) |
| Ti(1)-C(25)-H(25) | 118.8 | C(2)-Zr(1)-C(14) | 111.94(11) |
| C(25)-C(26)-C(27) | 108.3(3) | C(1)-Zr(1)-C(14) | 78.51(10) |
| C(25)-C(26)-Ti(1) | 73.81(19) | C(15)-Zr(1)-C(14) | 113.04(12) |
| C(27)-C(26)-Ti(1) | 73.11(18) | C(12)-Zr(1)-C(14) | 52.66(10) |
| C(25)-C(26)-H(26) | 125.9 | C(13)-Zr(1)-C(14) | 31.64(12) |
| C(27)-C(26)-H(26) | 125.9 | C(18)-Zr(1)-C(14) | 127.92(13) |
| Ti(1)-C(26)-H(26) | 119.1 | C(11)-Zr(1)-C(14) | 52.56(11) |
| C(26)-C(27)-C(28) | 107.4(3) | C(16)-Zr(1)-C(14) | 142.98(12) |
| C(26)-C(27)-Ti(1) | 72.5(2) | C(19)-Zr(1)-C(14) | 106.87(12) |
| C(28)-C(27)-Ti(1) | 74.6(2) | C(3)-Zr(1)-C(17) | 76.82(12) |
| C(26)-C(27)-H(27) | 126.3 | C(2)-Zr(1)-C(17) | 88.64(11) |
| C(28)-C(27)-H(27) | 126.3 | C(1)-Zr(1)-C(17) | 110.78(12) |
| Ti(1)-C(27)-H(27) | 118.6 | C(15)-Zr(1)-C(17) | 52.75(12) |
| C(29)-C(28)-C(27) | 108.1(3) | C(12)-Zr(1)-C(17) | 110.15(12) |
| C(29)-C(28)-Ti(1) | 74.1(2) | C(13)-Zr(1)-C(17) | 126.88(12) |
| C(27)-C(28)-Ti(1) | 71.3(2) | C(18)-Zr(1)-C(17) | 31.82(13) |
| C(29)-C(28)-H(28) | 125.9 | C(11)-Zr(1)-C(17) | 121.60(12) |
| C(27)-C(28)-H(28) | 125.9 | C(16)-Zr(1)-C(17) | 31.47(12) |
| Ti(1)-C(28)-H(28) | 120.4 | C(19)-Zr(1)-C(17) | 52.09(12) |
| C(28)-C(29)-C(25) | 108.5(3) | C(14)-Zr(1)-C(17) | 158.50(12) |

# 3 Synthesis of compounds

## 3.1 Synthesis of (Cp_2_Ti)(µ-Me)(µ-C_2_SiMe_3_)(ZrCp_2_) (7)

Cp_2_Ti(η^2^-Me_3_SiC_2_SiMe_3_) (**2**) (0.429 g, 1.22 mmol) and Cp_2_Zr(Me)(C_2_SiMe_3_) (**3**) (0.398 g, 1.23 mmol) were dissolved in 30 mL of benzene and the reaction mixture was stirred for 24 h at 60 °C. All volatile components were removed under vacuum and the remaining black greenish solid was dissolved in 40 mL of pentane. The resulting solution was filtered and slowly cooled to –78°C, resulting in the deposition of a dark greenish solid which was freed of its supernatant and dried under vacuum.

Note: Crystals suitable for single-crystal X-ray diffraction analysis were obtained by slow cooling of a toluene/pentane solution of **7** from room temperature to –78°C.

**Yield**: 0.139 g (23%).

**^1^H NMR** (300.13 MHz, C_6_D_6_, 297 K): δ 5.30 (s, 10 H, Cp); 4.94 (s, 10 H, Cp); 0.31 (s, ^2^*J*H,Si = 6.5 Hz, 9 H, Si*Me*_3_); –4.12 ppm (s, 3 H, *Me*). **^13^C{^1^H} NMR** (125.77 Hz, C_6_D_6_, 297 K): δ 276.6 (*C*CSiMe_3_); 167.3 (C*C*SiMe_3_); 105.5 (Cp); 101.6 (Cp); 9.4 (Zr*Me*); 1.8 ppm (Si*Me*_3_). **^29^Si-inept NMR** (99.35 MHz, C_6_D_6_, 24 °C): –11.7 ppm **MS** (CI^+^, isobutane): *m*/*z* (%) 510 (100) [Cp_2_Zr(CCSi(CH_3_)_3_TiCp_2_CH_3_]^+^, 495 (10) [M–Me]^+^, 414 (12) [Cp_2_Zr(CCSi(CH_3_)_3_]^+^, 333 (14) [M–TiCp_2_]^+^, 239 (3) [Cp_2_ZrMe]^+^, 178 (17) [Cp_2_Ti]^+^. **Mp**.: 157 °C (decomp.). **CHN analysis** (*Note: The compound class of dinuclear metallocene complexes shows a strong tendency to form ceramics during elemental analysis, which is why the measured values are reproducibly significantly lower than the theoretical values. To illustrate this, the best values obtained from three measurements are given here..*) calc. for C_26_H_32_SiTiZr: C, 61.03; H, 6.30. Found: C, 50.59; H, 5.67. **IR** (ATR, neat, cm^-1^): 381 (m), 465 (m), 585 (s), 661 (s), 736 (s), 838 (m), 1013 (m), 1128 (w), 1242 (w), 1440(w), 1719 (w), 2027 (vw), 2891 (w), 2952 (w), 3095 (vw).

**Figure S3.** ^1^H NMR spectrum (300.13 MHz, C_6_D_6_, 24 °C) of complex **7**. The spectrum was recorded from a sample which was recrystallised from pentane.

**Figure S4.** ^13^C{^1^H} NMR spectrum (125.77.6 MHz, C_6_D_6_, 24 °C) of complex **7**.

_._

**Figure S5.** ^29^Si-inept NMR spectrum (99.35 MHz, C_6_D_6_, 24 °C) of complex **7**.

## 3.2 Synthesis of (Cp_2_Ti)(µ-Me)(µ-C_2_Ph)(ZrCp_2_) (8)

Cp_2_Ti(η^2^-Me_3_SiC_2_SiMe_3_) (**2**) (0.208 g, 0.59 mmol) and Cp_2_Zr(Me)(C_2_Ph) (**3**) (0.200 g, 0.59 mmol) were dissolved in 5 mL of benzene and the reaction mixture was stirred for 24 h at 60 °C. All volatile components were removed under vacuum, and the remaining brownish solid was washed with 5 mL of pentane.

Note: Crystals suitable for single-crystal X-ray diffraction analysis were obtained by slow cooling of a saturated benzene solution of **8** from 60 °C to room temperature.

**Yield**: 0.100 g (33%).

**^1^H NMR** (400.13 MHz, C_6_D_6_, 297 K): δ 7.58-7.53 (m, 2 H, *o*-Ph); 7.42-7.35 (m, 2 H, *m*-Ph); 7.20 (m, 1 H, *p*-Ph); 5.35 (s, 10 H, Cp); 5.03 (s, 10 H, Cp); –3.72 ppm (s, 3 H, *Me*). **^13^C{^1^H} NMR** (100.63 Hz, C_6_D_6_, 297 K): δ 267.4 (Zr*C*CPh); 170.5 (ZrC*C*Ph); 140.2 (i-Ph); 130.5 (Ph); 128.8 (Ph); 126.1 (*p-*Ph); 105.5 (Cp); 102.4 (Cp); 8.4 ppm (*Me*). **MS** (CI^+^, isobutane): *m*/*z* (%) 514 (100) [Cp_2_Ti(CCPh)ZrCp_2_CH_3_]^+^, 499 (15) [M–Me]^+^, 337 (67) [M–Cp_2_Ti]^+^, 321 (25) [M–(Cp_2_TiMe)]^+^, 239 (32) [Cp_2_ZrMe]^+^, 193 (67) [Cp_2_TiMe]^+^, 178 (64) [Cp_2_Ti]^+^. **Mp**.: 206 °C (decomp.). **CHN analysis** (*Note: The compound class of dinuclear metallocene complexes shows a strong tendency to form ceramics during elemental analysis, which is why the measured values are reproducibly significantly lower than the theoretical values. To illustrate this, the best values obtained from three measurements are given here.*) calc. for C_29_H_28_TiZr: C, 67.55; H, 5.47. Found: C, 53.45; H, 5.07. **IR** (ATR, neat, cm^-1^): 450 (w), 492 (m), 547 (m), 569 (m), 602 (m), 652 (s), 692 (s), 752 (s), 796 (s), 1012 (m), 1066 (w), 1252 (vw), 1365 (vw), 1438 (w), 1479 (w), 1569 (w), 1590 (w), 1721 (m), 2946 (vw), 3085 (vw).

**Figure S6.** ^1^H NMR spectrum (400.13 MHz, C_6_D_6_, 24 °C) of complex **8**.

**Figure S7.** ^13^C{^1^H} NMR spectrum (100.6 MHz, C_6_D_6_, 24 °C) of complex **8**.

# 4 Computational details

Computational studies were carried out using *Gaussian16*^10^ and the standalone version of *NBO 6.0*.^[11]^ In this section, the main and secondary findings of a detailed DFT study are compiled to support the main findings discussed in the manuscript as well as to provide further detailed information. The real-size molecules were optimised using the hybrid density functional method B3LYP,^[12],^^[13]^ in combination with the basis set def2-TZVP,^[14]^ and Grimme’s empirical dispersion correction D3^[15]^ (notation: B3LYP-D3/def2-TZVP). Vibrational frequencies were computed, to include zero-point vibrational energies in thermodynamic parameters and to characterise all structures as minima on the potential energy surface. Using these geometries a series of binding analyses like Natural Bonding Orbital (NBO), Natural Localised Molecular Orbital (NLMO), Quantum-Theory of Atoms in Molecules (QT-AIM) and Wiberg bond index calculations/visualisation was performed. The latter two were performed using MultiWfn 3.5^[16]^, employing *Gaussian16* formatted checkpoint files.

For visualisation of 3D-quantum chemical results GaussView 6.1.1^[17]^ was used. In addition to these Supporting Information, a multi-structure xyz file with all calculated molecules is provided. For a better understanding and a more intuitive view of the calculated 3D structures, use of this file, e.g., with the free program MERCURY is recommended.^[18]^

*Please note that all computations were carried out for single, isolated molecules in the gas phase (ideal gas approximation). There may well be significant differences between gas phase and condensed phase.*

## 4.1 Thermodynamic computations

### 4.1.1 **Investigation of different isomers of complexes 7 and 8**

Since the zirconocene alkynyl methyl complex (**3**) served as the starting material in the synthesis of the heterodinuclear complexes, but we were able to observe the formation of titanocene σ-alkynyl complexes (**7** and **8**) using SC-XRD, this section investigates the energetic differences between four possible isomers. In addition to this manual isomer search, we also performed a conformer rotamer search (CREST)^[19]^ on complexes **7** and **8** for testing purposes. The geometries of the isomers identified in the SC-XRD analyses were specified as the initial connectivity. These analyses showed that, within a 50 kJ/mol energy window at the GFN2-xTB^[20]^ level of theory, 13 further conformers were found for complex **7** and eight for complex **8**, differing from the ground state only in minor structural changes, such as methyl and silyl group rotations for complex **7** and phenyl group rotations for complex **8**. Furthermore, isomers were found in which the bridging methyl group deviated more strongly from the reactive pocket. However, none of the analyses identified the transition to an isomer whose connectivity between the metal centre and the two bridging ligands (i.e., alkynyl and methyl) changes as had previously been analysed manually. It should be mentioned at this point that even after several attempts, the connectivity of **8** **iso2** could not be optimised as a stable isomer/intermediate. During these experiments, we only obtained the structure of **8 iso4** after geometry optimisation. Similar to the isomers of complex **7**, **8 iso4** also has significantly lower energy and thus always offers a sink into which the geometry is optimised.

### 4.1.2 **Theoretical investigation of different [Ph_3_C][B(C_6_F_5_)_4_] activation sequences.**

In this section, we analyse the first activation step of complexes **5**–**8** in reactions with the trityl salt [Ph_3_C][B(C_6_F_5_)_4_]. Two different sequences can occur in this first step: (1) cationic complexes and Ph_3_CCH_3_ can be formed, and (2) the formation of radical cationic species and Ph_3_C^•^ is conceivable.

**Table S4.** Summary of the theoretical investigation of the competing reaction pathways of the dinuclear complexes **5**–**8** towards trityl borate salt, forming either a cationic complex or a radical cationic complex (B3LYP-D3/def2-TZVP).

**Table S5.** Summary of the computed thermodynamic parameters of investigated compounds (B3LYP-D3/def2TZVP).

## 4.2 Bond analysis of isolated complexes

### 4.2.1 **Bond analysis of** **(Cp_2_Ti)(µ-Me)(µ-C_2_SiMe_3_)(ZrCp_2_) (7)**

#### 4.2.1.1 QT-AIM analysis of (Cp_2_Ti)(µ-Me)(µ-C_2_SiMe_3_)(ZrCp_2_) (**7**)

**Figure S8.** Contour plot of the Laplacian of the electron density ∇^2^*r* of complex **7** in the Ti1-Zr1-C3 plane. Dashed lines indicate negative (local charge concentration), solid lines indicate positive values (local charge depletion). The Laplacian plot is overlaid with the molecular graph from QT-AIM analysis and Wiberg bond indices (italic small numbers). Brown lines indicate bond paths, brown dashed lines are hypothetical bonds, blue dots correspond to bond critical points, light brown dots indicate ring critical points. Density from B3LYP-D3/def2-TZVP calculation.

#### 4.2.1.2 Spindensityplot of UHF Brokensymetry Wavefunktion of (Cp_2_Ti)(µ-Me)(µ-C_2_SiMe_3_)(ZrCp_2_) (**7**)

**Figure S9.** Spindensity plot of UHF broken symmetry wave function of (Cp_2_Ti)(µ-Me)(µ-C_2_SiMe_3_)(ZrCp_2_) (**7**) from HF/def2-TZVP stable = opt calculation. (Titanium left, zirconium right).

```
Sum of Mulliken charges =   0.00000  -0.00000
 Mulliken charges and spin densities with hydrogens summed into heavy atoms:
               1          2
     1  C   -0.253803  -0.068708
     2  Zr   0.977242   0.734504
     3  Ti   0.570887  -1.074497
     7  C   -0.064616   0.558947
     8  C   -0.452984  -0.236693
     9  Si   0.484727   0.034384
    10  C   -0.119291   0.000730
    14  C   -0.125286  -0.001828
    18  C   -0.119714  -0.002823
    22  C   -0.031515  -0.005435
    23  C   -0.074584   0.035053
    24  C   -0.000804   0.010093
    26  C   -0.110762   0.010291
    28  C    0.011946   0.009938
    32  C   -0.043796  -0.012995
    33  C   -0.091369  -0.011220
    34  C    0.005121  -0.015201
    36  C   -0.031786   0.018309
    38  C   -0.088943  -0.009896
    42  C   -0.035455   0.002876
    43  C   -0.074170   0.034787
    44  C   -0.139373  -0.012487
    45  C    0.060655  -0.016862
    46  C   -0.019934   0.004031
    48  C    0.013189  -0.000287
    50  C   -0.085164   0.016797
    54  C   -0.074708   0.014555
    55  C   -0.101337  -0.017638
    58  C    0.015625   0.001276
```

#### 4.2.1.3 Highest Molecular Orbital of (Cp_2_Ti)(µ-Me)(µ-C_2_SiMe_3_)(ZrCp_2_) (**7**)

**Figure S10.** HOMO plot of (Cp_2_Ti)(µ-Me)(µ-C_2_SiMe_3_)(ZrCp_2_) (**7**) from closed shell singlet solution at B3LYP-D3/def2-TZVP level of theory. (isovalue: 0.003; E = - 4.521 eV).

### 4.2.2 **Bond analysis of (Cp_2_Ti)(µ-Me)(µ-C_2_Ph)(ZrCp_2_) (8)**

#### 4.2.2.1 QT-AIM analysis of (Cp_2_Ti)(µ-Me)(µ-C_2_Ph)(ZrCp_2_) (**8**)

**Figure S11.** Contour plot of the Laplacian of the electron density ∇^2^*r* of complex **8** in the Ti1-Zr1-C3 plane. Dashed lines indicate negative (local charge concentration), solid lines indicate positive values (local charge depletion). The Laplacian plot is overlaid with the molecular graph from QT-AIM analysis and Wiberg bond indices (italic small numbers). Brown lines indicate bond paths, brown dashed lines are hypothetical bonds, blue dots correspond to bond critical points, light brown dots indicate ring critical points. Density from B3LYP-D3/def2-TZVP calculation.

#### 4.2.2.2 Spindensityplot of UHF Brokensymetry Wavefunktion of (Cp_2_Ti)(µ-Me)(µ-C_2_Ph)(ZrCp_2_) (**8**)

**Figure S12.** Spindensity plot of UHF broken symmetry wave function of (Cp_2_Ti)(µ-Me)(µ-C_2_Ph)(ZrCp_2_) (**8**) from HF/def2-TZVP stable = opt calculation. (Titanium left, zirconium right).

```
Sum of Mulliken charges =   0.00000   0.00000
 Mulliken charges and spin densities with hydrogens summed into heavy atoms:
               1          2
     1  C   -0.241432  -0.057842
     2  Zr   0.965591   0.678414
     3  Ti   0.586472  -1.130896
     7  C    0.103840   1.032166
     8  C   -0.430934  -0.589465
     9  C    0.115628   0.599168
    10  C   -0.096738  -0.563790
    11  C   -0.128359  -0.578986
    12  C    0.003752   0.543617
    14  C    0.002435  -0.566933
    16  C   -0.011856   0.550138
    20  C   -0.063379   0.013448
    21  C    0.069397  -0.025209
    22  C   -0.112466  -0.016619
    24  C   -0.125031  -0.004182
    26  C    0.015271   0.001541
    29  C   -0.027257   0.010209
    30  C    0.002186  -0.007111
    31  C   -0.095078  -0.005023
    33  C   -0.053406  -0.010283
    35  C   -0.058419  -0.019155
    39  C   -0.086785   0.011423
    40  C   -0.019192   0.014959
    41  C    0.012756   0.004028
    43  C   -0.036966   0.001590
    45  C   -0.078487   0.040298
    49  C    0.008583   0.023610
    50  C   -0.113860   0.005138
    51  C   -0.073838   0.032205
    53  C   -0.044623  -0.003835
    55  C    0.012196   0.017377
```

#### 4.2.2.3 Highest Molecular Orbital of (Cp_2_Ti)(µ-Me)(µ-C_2_Ph)(ZrCp_2_) (**8**)

**Figure S13.** HOMO plot of (Cp_2_Ti)(µ-Me)(µ-C_2_Ph)(ZrCp_2_) (**8**) from closed shell singlet solution at B3LYP-D3/def2-TZVP level of theory. (isovalue: 0.003; E = - 4.739 eV).

## 4.3 Bond analysis of hypothetically formed cationic complexes

**Table S6.** Summary of selected Wiberg Bond Indices from the computed hypothetical activated metallocene fragments.

### 4.3.1 **Bond analysis of hypothetically formed [(Cp_2_Zr)(µ-C_2_SiMe_3_)(ZrCp_2_)] (5^+^)**

**Figure S14.** Representation of the optimised geometry of hypothetically formed cationic complex **5^+^** and labelling of the atoms in computational investigation. Hydrogens are omitted for clarity.

#### 4.3.1.1 QT-AIM analysis of [(Cp_2_Zr)(µ-C_2_SiMe_3_)(ZrCp_2_)] (**5^+^**)

**Figure S15.** Contour plot of the Laplacian of the electron density ∇^2^*r* of complex **5^+^** in the Zr27-Zr11-C25 plane. Dashed lines indicate negative (local charge concentration), solid lines indicate positive values (local charge depletion). The Laplacian plot is overlaid with the molecular graph from QT-AIM analysis and Wiberg bond indices (italic small numbers). Brown lines indicate bond paths, brown dashed lines are hypothetical bonds, blue dots correspond to bond critical points, light brown dots indicate ring critical points. Density from B3LYP-D3/def2-TZVP calculation.

### 4.3.2 **Bond analysis of hypothetically formed [(Cp_2_Zr)(µ-C_2_Ph)(ZrCp_2_)] (6^+^)**

**Figure S16.** Representation of the optimised geometry of hypothetically formed cationic complex **6^+^** and labelling of the atoms in computational investigation. Hydrogens are omitted for clarity.

#### 4.3.2.1 QT-AIM analysis of [(Cp_2_Zr)(µ-C_2_Ph)(ZrCp_2_)] (**6^+^**)

**Figure S17.** Contour plot of the Laplacian of the electron density ∇^2^*r* of complex **6^+^** in the Zr55-Zr54-C2 plane. Dashed lines indicate negative (local charge concentration), solid lines indicate positive values (local charge depletion). The Laplacian plot is overlaid with the molecular graph from QT-AIM analysis and Wiberg bond indices (italic small numbers). Brown lines indicate bond paths, brown dashed lines are hypothetical bonds, blue dots correspond to bond critical points, light brown dots indicate ring critical points. Density from B3LYP-D3/def2-TZVP calculation.

### 4.3.3 **Bond analysis of hypothetically formed [(Cp_2_Ti)(µ-C_2_SiMe_3_)(ZrCp_2_)] (7^+^)**

**Figure S18.** Representation of the optimised geometry of hypothetically formed cationic complex **7^+^** and labelling of the atoms in computational investigation. Hydrogens are omitted for clarity.

#### 4.3.3.1 QT-AIM analysis of [(Cp_2_Ti)(µ-C_2_SiMe_3_)(ZrCp_2_)] (**7^+^**)

**Figure S19.** Contour plot of the Laplacian of the electron density ∇^2^*r* of complex **7^+^** in the Ti2-Zr1-C4 plane. Dashed lines indicate negative (local charge concentration), solid lines indicate positive values (local charge depletion). The Laplacian plot is overlaid with the molecular graph from QT-AIM analysis and Wiberg bond indices (italic small numbers). Brown lines indicate bond paths, brown dashed lines are hypothetical bonds, blue dots correspond to bond critical points, light brown dots indicate ring critical points. Density from B3LYP-D3/def2-TZVP calculation.

### 4.3.4 **Bond analysis of hypothetically formed [(Cp_2_Ti)(µ-C_2_Ph)(ZrCp_2_)] (8^+^)**

**Figure S20.** Representation of the optimised geometry of hypothetically formed cationic complex **8^+^** and labelling of the atoms in computational investigation. Hydrogens are omitted for clarity.

#### 4.3.4.1 QT-AIM analysis of [(Cp_2_Ti)(µ-C_2_Ph)(ZrCp_2_)] (**8^+^**)

**Figure S21.** Contour plot of the Laplacian of the electron density ∇^2^*r* of complex **8^+^** in the Ti2-Zr1-C4 plane. Dashed lines indicate negative (local charge concentration), solid lines indicate positive values (local charge depletion). The Laplacian plot is overlaid with the molecular graph from QT-AIM analysis and Wiberg bond indices (italic small numbers). Brown lines indicate bond paths, brown dashed lines are hypothetical bonds, blue dots correspond to bond critical points, light brown dots indicate ring critical points. Density from B3LYP-D3/def2-TZVP calculation.

### 4.3.5 **Bond analysis of hypothetically formed [(Cp_2_Zr)(µ-Me)(µ-C_2_SiMe_3_)(ZrCp_2_)] (5^•+^)**

**Figure S22.** Representation of the optimised geometry of hypothetically formed cationic complex **5^•+^** and labelling of the atoms in computational investigation. Hydrogens are omitted for clarity.

#### 4.3.5.1 QT-AIM analysis of [(Cp_2_Zr)(µ-C_2_SiMe_3_)(ZrCp_2_)] (**5^•+^**)

**Figure S23.** Contour plot of the Laplacian of the electron density ∇^2^*r* of complex **5^•+^** in the Zr28-Zr12-C26 plane. Dashed lines indicate negative (local charge concentration), solid lines indicate positive values (local charge depletion). The Laplacian plot is overlaid with the molecular graph from QT-AIM analysis and Wiberg bond indices (italic small numbers). Brown lines indicate bond paths, brown dashed lines are hypothetical bonds, blue dots correspond to bond critical points, light brown dots indicate ring critical points. Density from B3LYP-D3/def2-TZVP calculation.

### 4.3.6 **Bond analysis of hypothetically formed [(Cp_2_Zr)(µ-C_2_Ph)(ZrCp_2_)] (6^•+^)**

**Figure S24.** Representation of the optimised geometry of hypothetically formed cationic complex **6^•+^** and labelling of the atoms in computational investigation. Hydrogens are omitted for clarity.

#### 4.3.6.1 QT-AIM analysis of [(Cp_2_Zr)(µ-C_2_Ph)(ZrCp_2_)] (**6^•+^**)

**Figure S25.** Contour plot of the Laplacian of the electron density ∇^2^*r* of complex **6^•+^** in the Zr56-Zr55-C3 plane. Dashed lines indicate negative (local charge concentration), solid lines indicate positive values (local charge depletion). The Laplacian plot is overlaid with the molecular graph from QT-AIM analysis and Wiberg bond indices (italic small numbers). Brown lines indicate bond paths, brown dashed lines are hypothetical bonds, blue dots correspond to bond critical points, light brown dots indicate ring critical points. Density from B3LYP-D3/def2-TZVP calculation.

### 4.3.7 **Bond analysis of hypothetically formed [(Cp_2_Ti)(µ-C_2_SiMe_3_)(ZrCp_2_)] (7^•+^)**

**Figure S26.** Representation of the optimised geometry of hypothetically formed cationic complex **7^•+^** and labelling of the atoms in computational investigation. Hydrogens are omitted for clarity.

#### 4.3.7.1 QT-AIM analysis of [(Cp_2_Ti)(µ-C_2_SiMe_3_)(ZrCp_2_)] (**7^•+^**)

**Figure S27.** Contour plot of the Laplacian of the electron density ∇^2^*r* of complex **7^•+^** in the Ti3-Zr2-C8 plane. Dashed lines indicate negative (local charge concentration), solid lines indicate positive values (local charge depletion). The Laplacian plot is overlaid with the molecular graph from QT-AIM analysis and Wiberg bond indices (italic small numbers). Brown lines indicate bond paths, brown dashed lines are hypothetical bonds, blue dots correspond to bond critical points, light brown dots indicate ring critical points. Density from B3LYP-D3/def2-TZVP calculation.

### 4.3.8 **Bond analysis of hypothetically formed [(Cp_2_Ti)(µ-C_2_Ph)(ZrCp_2_)] (8^•+^)**

**Figure S28.** Representation of the optimised geometry of hypothetically formed cationic complex **8^•+^** and labelling of the atoms in computational investigation. Hydrogens are omitted for clarity.

#### 4.3.8.1 QT-AIM analysis of [(Cp_2_Ti)(µ-C_2_Ph)(ZrCp_2_)] (**8^•+^**)

**Figure S29.** Contour plot of the Laplacian of the electron density ∇^2^*r* of complex **8^•+^** in the Ti2-Zr1-C4 plane. Dashed lines indicate negative (local charge concentration), solid lines indicate positive values (local charge depletion). The Laplacian plot is overlaid with the molecular graph from QT-AIM analysis and Wiberg bond indices (italic small numbers). Brown lines indicate bond paths, brown dashed lines are hypothetical bonds, blue dots correspond to bond critical points, light brown dots indicate ring critical points. Density from B3LYP-D3/def2-TZVP calculation.

### 4.3.9 **Bond analysis of hypothetically formed [(Cp_2_Zr)(µ-C_2_SiMe_3_)(ZrCp_2_)] (5^•^)**

**Figure S30.** Representation of the optimised geometry of hypothetically formed cationic complex **5^•^** and labelling of the atoms in computational investigation. Hydrogens are omitted for clarity.

#### 4.3.9.1 QT-AIM analysis of [(Cp_2_Zr)(µ-C_2_SiMe_3_)(ZrCp_2_)] (**5^•^**)

**Figure S31.** Contour plot of the Laplacian of the electron density ∇^2^*r* of complex **5^•^** in the Zr27-Zr11-C25 plane. Dashed lines indicate negative (local charge concentration), solid lines indicate positive values (local charge depletion). The Laplacian plot is overlaid with the molecular graph from QT-AIM analysis and Wiberg bond indices (italic small numbers). Brown lines indicate bond paths, brown dashed lines are hypothetical bonds, blue dots correspond to bond critical points, light brown dots indicate ring critical points. Density from B3LYP-D3/def2-TZVP calculation.

### 4.3.10 **Bond analysis of hypothetically formed [(Cp_2_Zr)(µ-C_2_Ph)(ZrCp_2_)] (6^•^)**

**Figure S32.** Representation of the optimised geometry of hypothetically formed cationic complex **6^•^** and labelling of the atoms in computational investigation. Hydrogens are omitted for clarity.

#### 4.3.10.1 QT-AIM analysis of [(Cp_2_Zr)(µ-C_2_Ph)(ZrCp_2_)] (**6^•^**)

**Figure S33.** Contour plot of the Laplacian of the electron density ∇^2^*r* of complex **6^•^** in the Zr55-Zr54-C2 plane. Due to the bending of the central unit, the C1 atom is not in the same plane, so the bond paths to C1 are not fully represented. Dashed lines indicate negative (local charge concentration), solid lines indicate positive values (local charge depletion). The Laplacian plot is overlaid with the molecular graph from QT-AIM analysis and Wiberg bond indices (italic small numbers). Brown lines indicate bond paths, brown dashed lines are hypothetical bonds, blue dots correspond to bond critical points, light brown dots indicate ring critical points. Density from B3LYP-D3/def2-TZVP calculation.

### 4.3.11 **Bond analysis of hypothetically formed [(Cp_2_Ti)(µ-C_2_SiMe_3_)(ZrCp_2_)] (7^•^)**

**Figure S34.** Representation of the optimised geometry of hypothetically formed cationic complex **7^•^** and labelling of the atoms in computational investigation. Hydrogens are omitted for clarity.

#### 4.3.11.1 QT-AIM analysis of [(Cp_2_Ti)(µ-C_2_SiMe_3_)(ZrCp_2_)] (7^•^)

**Figure S35.** Contour plot of the Laplacian of the electron density ∇^2^*r* of complex **7^•^** in the Zr1-Ti2-C4 plane. Dashed lines indicate negative (local charge concentration), solid lines indicate positive values (local charge depletion). The Laplacian plot is overlaid with the molecular graph from QT-AIM analysis and Wiberg bond indices (italic small numbers). Brown lines indicate bond paths, brown dashed lines are hypothetical bonds, blue dots correspond to bond critical points, light brown dots indicate ring critical points. Density from B3LYP-D3/def2-TZVP calculation.

### 4.3.12 **Bond analysis of hypothetically formed [(Cp_2_Ti)(µ-C_2_Ph)(ZrCp_2_)] (8^•^)**

**Figure S36.** Representation of the optimised geometry of hypothetically formed cationic complex **8^•^** and labelling of the atoms in computational investigation. Hydrogens are omitted for clarity.

#### 4.3.12.1 QT-AIM analysis of [(Cp_2_Ti)(µ-C_2_Ph)(ZrCp_2_)] (**8^•^**)

**Figure S37.** Contour plot of the Laplacian of the electron density ∇^2^*r* of complex **8^•^** in the Zr1-Ti2-C4 plane. Dashed lines indicate negative (local charge concentration), solid lines indicate positive values (local charge depletion). The Laplacian plot is overlaid with the molecular graph from QT-AIM analysis and Wiberg bond indices (italic small numbers). Brown lines indicate bond paths, brown dashed lines are hypothetical bonds, blue dots correspond to bond critical points, light brown dots indicate ring critical points. Density from B3LYP-D3/def2-TZVP calculation.

### 4.3.13 **Natural charge and spin analysis of hypothetically formed cationic complexes**

This section summarises the natural charge and spin distributions in the investigated cationic and radical cationic complexes based on NBO6.0 analysis. The complexes were divided into three units: Cp_2_M1, Cp_2_M2 and the remaining atoms.

#### 4.3.13.1 Natural charge distribution in hypothetical cation **5^+^**

#### 4.3.13.2 Natural charge distribution in hypothetical cation **6^+^**

#### 4.3.13.3 Natural charge distribution in hypothetical cation **7^+^**

#### 4.3.13.4 Natural charge distribution in hypothetical cation **8^+^**

#### 4.3.13.5 Natural charge and spin distribution in hypothetical radical cation **5^•+^**

#### 4.3.13.6 Natural charge and spin distribution in hypothetical radical cation **6^•+^**

#### 4.3.13.7 Natural charge and spin distribution in hypothetical radical cation **7^•+^**

#### 4.3.13.8 Natural charge and spin distribution in hypothetical radical cation **8^•+^**

#### 4.3.13.9 Natural charge and spin distribution in hypothetical radical **5^•^** without bridging methyl ligand

#### 4.3.13.10 Natural charge and spin distribution in hypothetical radical **6^•^** without bridging methyl ligand

#### 4.3.13.11 Natural charge and spin distribution in hypothetical radical **7^•^** without bridging methyl ligand

#### 4.3.13.12 Natural charge and spin distribution in hypothetical radical **8^•^** without bridging methyl ligand

### 4.3.14 **Spin density analysis of hypothetically formed radical cationic complexes**

#### 4.3.14.1 Representation of the spin density in the radical cation **5^•+^**

**Figure S38.** Representation of the spin density plot in the radical cation [(Cp_2_Zr)(µ-Me)(µ-C_2_SiMe_3_)(ZrCp_2_)]^•+^ (**5****^•+^**) (UB3LYP-D3/def2-TZVP, isovalue 0.01).

#### 4.3.14.2 Representation of the spin density in the radical cation **6^•+^**

**Figure S39.** Representation of the spin density plot in the radical cation [(Cp_2_Zr)(µ-Me)(µ-C_2_Ph)(ZrCp_2_)]^•+^ (**6^•+^**) (UB3LYP-D3/def2-TZVP, isovalue 0.01).

#### 4.3.14.3 Representation of the spin density in the radical cation **7^•+^**

**Figure S40.** Representation of the spin density plot in the radical cation [(Cp_2_Ti)(µ-Me)(µ-C_2_SiMe_3_)(ZrCp_2_)]^•+^ (**7^•+^**) (UB3LYP-D3/def2-TZVP, isovalue 0.01).

#### 4.3.14.4 Representation of the spin density in the radical cation **8^•+^**

**Figure S41.** Representation of the spin density plot in the radical cation [(Cp_2_Ti)(µ-Me)(µ-C_2_Ph)(ZrCp_2_)]^•+^ (**8^•+^**) (UB3LYP-D3/def2-TZVP, isovalue 0.01).

#### 4.3.14.5 Representation of the spin density in the radical **5^•^** without bridging methyl ligand

**Figure S42.** Representation of the spin density plot in the radical [(Cp_2_Zr)(µ-C_2_SiMe_3_)(ZrCp_2_)]^•^ (**5^•^**) (UB3LYP-D3/def2-TZVP, isovalue 0.01).

#### 4.3.14.6 Representation of the spin density in the radical **6^•^** without bridging methyl ligand

**Figure S43.** Representation of the spin density plot in the radical [(Cp_2_Zr)(µ-C_2_Ph)(ZrCp_2_)]^•^ (**6^•^**) (UB3LYP-D3/def2-TZVP, isovalue 0.01).

#### 4.3.14.7 Representation of the spin density in the radical **7^•^** without bridging methyl ligand

**Figure S44.** Representation of the spin density plot in the radical [(Cp_2_Ti)(µ-C_2_SiMe_3_)(ZrCp_2_)]^•^ (**7^•^**) (UB3LYP-D3/def2-TZVP, isovalue 0.01).

#### 4.3.14.8 Representation of the spin density in the radical **8^•^** without bridging methyl ligand

**Figure S45.** Representation of the spin density plot in the radical [(Cp_2_Ti)(µ-C_2_Ph)(ZrCp_2_)]^•^ (**8^•^**) (UB3LYP-D3/def2-TZVP, isovalue 0.01).

# 5 Ethylene polymerisation experiments

Homo- and heterobimetallic complexes Cp_2_Zr(μ-CH_3_)(μ-CCSiMe_3_)ZrCp_2_ (**5**), Cp_2_Zr(μ-CH_3_)(μ-CCPh)ZrCp_2_ (**6**), Cp_2_Ti(μ-CH_3_)(μ-CCSiMe_3_)ZrCp_2_ (**7**), Cp_2_Ti(μ-CH_3_)(μ-CCPh)ZrCp_2_ (**8**), and the mononuclear precursor complexes Cp_2_Zr(CCSiMe_3_)(CH_3_) (**3**) and Cp_2_Zr(CCPh)(CH_3_) (**4**) were tested in ethylene polymerisation in the presence of aluminum-free organoboron activators, namely [Ph_3_C][B(C_6_F_5_)_4_] (TBT), B(C_6_F_5_)_3_ and a mixture of B(C_6_F_5_)_3_/Et_3_SiH (SiHB). Polymerisation reactions were carried out in a semi-batch mode using a 250 mL double-jacketed glass autoclave. Active species were generated *in situ* using an equimolar ratio of organoboron compound to precatalyst (sub-stoichiometric to metals for the binuclear complexes).

The primary objective of the catalytic evaluation of dinuclear complexes **5**–**8** was to compare their relative activities in ethylene polymerisation. However, preliminary experiments revealed substantial differences in catalytic activity across the series. To obtain measurable polymer yields for the less active systems, it was necessary to employ relatively high precatalyst concentrations. For consistency, reference systems (Cp_2_ZrMe_2_ and Cp_2_TiMe_2_) were evaluated under identical reaction conditions. It should be noted that the catalytic systems without a scavenger typically exhibit a sharp decrease of activity when decreasing the catalyst concentration under a certain level. For Cp_2_ZrMe_2_, we reached very similar activities for the borane-activated system (expressed as relative to ethylene pressure) as Marks et al.^[21]^, while they ran the polymerisations at lower ethylene pressure (1 bar vs 3 bar), which led to narrower *M_w_*/*M_n_* (around 2) of the polymers than in the present case. Cp_2_ZrCl_2_ polymerisation activated by the SiHB system was studied in our previous work^[22]^ and the PE produced had also a narrower dispersity in comparison to the herein described Cp_2_ZrMe_2_.

Under our conditions, highly active systems indeed generated significant heat release during the polymerisation, which would require further optimisation. Even at short reaction times, a pronounced temperature increase was observed due to the strongly exothermic nature of ethylene insertion (*T*_2_ values in tables). Thus, we believe that two main factors contributed to the observed broadening of molar mass dispersity (*M_w_*/*M_n_*). For the thermally robust system Cp_2_ZrMe_2_, the increase in dispersity is attributed primarily to enhanced β-hydride elimination at elevated temperatures, which increases the frequency of chain-transfer and termination events. This fact has additionally been mentioned in the Results and Discussion section of the manuscript.

In contrast, the Cp_2_TiMe_2_ system (*M_w_*/*M_n_* = 11.9) exhibits substantially broader dispersity despite much lower absolute activity. We propose that thermally induced transformation (thermally induced titanocene bis-alkyl transformations are well known and were studied by us and others) of the active species leads to the formation of two distinct catalytic centres with different propagation kinetics. This results in a bimodal molar mass distribution, as evidenced by the GPC traces (Figure S48, Table S9, last entry).

## 5.1 General procedure for ethylene polymerisation

All polymerisation reactions were performed in a 250 mL Büchi glass double-jacketed laboratory autoclave equipped with a magnetic propeller-like stirrer at constant ethylene pressure. The hot autoclave was evacuated three times and filled with Ar, charged with toluene, and the desired amount of activator (from a 1.6 **×** 10^–3^ M stock solution of [Ph_3_C][B(C_6_F_5_)_4_]). The inert gas was vented, and the reactor was pressurised with ethylene for 10 min. Then, the ethylene pressure was partially released, and the polymerisation was started by injecting the desired amount of precatalyst in toluene under a stream of ethylene (fresh solution in toluene was used for each experiment, prepared just before loading into the autoclave). The same procedure was used for the binary activator (Et_3_SiH/B(C_6_F_5_)_3_), except that the mixture of solvent (toluene or dichloromethane), silane and borane was stirred for 30 min before catalyst loading. The final volume of the polymerisation solution was 50 mL for all experiments. The autoclave temperature was monitored during the reaction by using an external Pt100 sensor connected to a Julabo F31-C bath. Data collection was performed for temperature changes inside the reactor as well as for ethylene uptake during the polymerisation. The consumption of ethylene was followed in each experiment using a calibrated mass flow meter (Bronkhorst, EL-FLOW, 1L/min). After the appropriate time, the reactor was vented and the residual mixture in autoclave was quenched with 80 mL of methanol. The precipitated polymer was stirred for 1 h, filtered off on a glass frit, rinsed repeatedly with methanol and acetone, and dried in vacuum to constant weight.

### 5.1.1 **Polyethylene characterisation**

Molar masses and dispersities of the polymer samples were determined using a HT SEC chromatograph (PL GPC 220 high temperature chromatograph equipped with PL-220DRI and VISKOTEL 220R detectors).

FTIR spectra of polymers were measured on a Nicolet iS10 FT-IR instrument. Transmission spectra were registered in the region 4000 – 400 cm-^1^ using 64 scans at a resolution of 4 cm^-1^. Solid samples of polymers were pressed on a special press at laboratory temperature into thin foils with a thickness optimally in the range of 200-500 µm and directly analysed in the transmission mode of the spectrometer.

The foil thickness was determined from the average of three measurements using a digital micrometer. For silyl-terminated polymers, the number of Et_3_Si groups per 1000 carbons of the polymer chain (N_Si_= Si/1000C) was estimated by using the molar absorption coefficient (ϵ) of signal at 1017 cm^-1^ determined for 1-triethylsilyltetradecane. The absorption coefficient of 1-triethylsilyltetradecane was determined from a solution of a sample of known concentration in hexane (four experiments at different conc.) using the Lambert-Beer relation ϵ= A c^-1^t^-1^, where *A* is the measured signal absorbance at a wavelength of 1017 cm^-1^, *c* is the sample concentration and *t* denotes the thickness of the cuvette used. The exact cuvette thickness (KBr) was determined from the interference signals of the empty cuvette (t= 0.109 mm). To calculate N_Si_, the relation of N_Si_= 14 A (ρ t ϵ)^-1^ was used, where A is the absorbance of the signal at 1016 cm^-1^, *ρ* is the density of the polymer (0.95 g m L^-1^), *t* is the thickness of the PE film (cm) and *ϵ* denotes the molar absorption coefficient of 1-triethylsilyltetradecane ϵ= 98 (5) L mol^−1^ cm^−1^.

For the quantitative estimation of unsaturated groups in polymer products, the molar absorption coefficients of signals 908 cm^-1^ for the vinyl group in 1-dodecene (ε_908_ = 110 L mol^-1^ cm^-1^), 964 cm^-1^ for trans-vinylene (ε_964_ = 168 L mol^-1^ cm^-1^), and 888 cm^-1^ for vinylidene in 2,3-dimethylbutadiene (ε_888_ = 146 L mol^-1^ cm^-1^) were used.^[23]^

## 5.2 Polymerisation reactions with [Ph_3_C][B(C_6_F_5_)_4_] (TBT) as the activator

### 5.2.1 **System optimisation**

Initial experiments with the **6**/TBT system showed that ethylene polymerisation occurs immediately after the injection of precatalyst into the reactor (preloaded with solvent and co-catalyst, and presaturated with ethylene at a given pressure) to produce large amounts of precipitated polymer and reactor fouling within seconds. Brief optimisation of the experimental conditions (Table S7), to diminish mass transport and diffusion issues, resulted in a minimum catalyst concentration of 1.5 x 10^-4^ mol L^-1^ (at 1 bar of ethylene pressure) with an acceptable experimental reproducibility. However, even under these conditions, the reaction is highly exothermic (Table S7, run 3), the temperature rises rapidly from 25 ^o^C to 39 ^o^C in less than a minute with loss of control during the polymerisation. This should be taken into account when comparing the properties of obtained polymers, particularly molar masses and dispersities. Further decrease in catalyst concentration resulted to non-reproducible polymer yields and finally to an inactive system at concentration of 1 x 10^-4^ mol/L (Table S7, run 4).

**Table S7. 6**/TBT catalyst optimisation. *^a^*

| Run | Cat. | Cat.  *(µmol)* | p  *(bar)* | t  *(sec)* | T_2_  *(^0^C)* | PE  *(g)* | A*^b^* | M_n_  *(g/mol)* | M_w_/M_n_ | Notes^c^ |
| --- | --- | --- | --- | --- | --- | --- | --- | --- | --- | --- |
| 1 | **6** | 15 | 3 | 23 | 45 | 1.520 | 5 290 | 96 900 | 3.28 | green |
| 2 | **6** | 7.5 | 3 | 22 | 42 | 1.090 | 7 930 | 141 000 | 3.48 | green |
| 3 | **6** | 7.5 | 1 | 55 | 39 | 0.885 | 7 720 | 196 400 | 3.25 | green→gray |
| 4 | **6** | 5 | 1 | 600 | 25 | 0 | 0 | - | - | gray |

*^a^ Polymerisation conditions:* total volume 50 mL, ratio TBT/**6**: 1.1, T_1_ = starting temperature 25 ^o^C, T_2_ = final temperature just before quenching, rpm 800. *^b^* A= kg_PE_ (mol_cat_ bar h)^-1^. ^c^ Colour of the mixture after initiation.

### 5.2.2 **Ethylene polymerisation results initiated by TBT systems**

**Table S8.** Ethylene polymerisation results initiated by TBT systems.*^a^*

| Cat. | t  *(sec)* | T_2_  *(^0^C)* | PE  *(g)* | A*^b^* | M_n_  *(g/mol)* | M_w_/M_n_ | Notes^d^ |
| --- | --- | --- | --- | --- | --- | --- | --- |
| **5** | 613 | 25 | *trace* | *trace* |  |  | deep blue |
| **6** | 55 | 39 | 0.885 | 7 720 | 196 400 | 3.25 | green→gray |
| **7** | 314 | 29 | 0.325 | 500 | 139 100 | 3.01 | yellow |
| **8** | 48 | 39 | 0.874 | 8 740 | 138 500 | 3.76 | green |
| **3** | 339 | 25 | *trace* | *trace* |  |  | orange |
| **4** | 315 | 25 | *trace* | *trace* |  |  | yellow |
| **4^c^** | 338 | 25 | 0.138 | 100 | 73 700 | 4.34 | orange |
| Cp_2_ZrMe_2_ | 56 | 40 | 1.083 | 9 280 | 140 200 | 4.13 | orange |

*^a^ Polymerisation conditions:* total volume 50 mL, polymerisation medium toluene, cat. dissolved in toluene, pressure 1 bar, T_1_ = starting temperature 25 ^o^C, T_2_ = final temperature just before quenching, rpm 800, catalyst 7.5 μmol, ratio TBT/catalyst = 1.1. *^b^* A = kg_PE_ (mol_cat_ bar h)^-1^. ^c^ catalyst 15 μmol. ^d^ Colour of the mixture after initiation.

Total unsaturation of polymer initiated by TBT systems:
catalyst (**6**) PE ∑(C=C)/1000C= 0.047 (65%)
catalyst (**8**) PE ∑(C=C)/1000C= 0.061 (60%)
catalyst (**7**) PE ∑(C=C)/1000C= 0.033 (33%)
catalyst (Cp_2_ZrMe_2_) PE ∑(C=C)/1000C= 0.046 (46%)

**Figure S46.** FT-IR spectra (PE foils, full region) of polymers obtained from ethylene polymerisation results initiated by TBT systems.

**Figure S47.** FT-IR spectra (PE foils, an expanded region) of polymers obtained from ethylene polymerisation results initiated by TBT systems. Bands at 908 and 980 cm^−1^ are characteristic of vinyl vibrations, while the band at 966 cm^-1^ is suggested to be characteristic of internal double bonds.

## 5.3 Polymerisation reactions with B(C_6_F_5_)_3_ and B(C_6_F_5_)_3_/Et_3_SiH as the activators

**Table S9.** Ethylene polymerisation results initiated by B(C_6_F_5_)_3_ systems. *^a^*

| Cat. | t  *(sec)* | T_2_  *(^0^C)* | PE  *(g)* | A | M_n_  *(g/mol)* | M_w_/M_n_ | Notes^b^ |
| --- | --- | --- | --- | --- | --- | --- | --- |
| **5** | 201 | 29 | 0.340 | 140 | 103 000 | 3.31 | orange |
| **6** | 198 | 42 | 1.692 | 680 | 159 000 | 2.95 | yellow |
| **7** | 76 | 45 | 1.803 | 1 900 | 141 000 | 3.28 | violet→yellow |
| **8** | 85 | 43 | 1.240 | 1 170 | 134 900 | 3.29 | deep yellow |
| **3** | 480 | 25 | *trace* | *trace* |  |  | slightly yellow |
| **4** | 365 | 28 | 0.210 | 50 | 93 000 | 4.66 | yellow |
| Cp_2_ZrMe_2_ | 41 | 56 | 2.034 | 3 970 | 107 300 | 3.15 | colourless |
| Cp_2_TiMe_2_ | 610 | 34 | 0.220 | 30 | 15 700 | 11.9 | yellow*, bimodal* |

*^a^ Polymerisation conditions:* total volume 50 mL, polymerisation medium toluene, cat. dissolved in toluene, pressure 3 bar, T_1_ = starting temperature 25 ^o^C, T_2_ = final temperature before quenching, rpm 800, catalyst 15 μmol, ratio B(C_6_F_5_)_3_/catalyst = 1.1. A = kg_PE_ (mol_cat_ bar h)^-1^. ^b^ Colour of the mixture after initiation.

**Figure S48.** *M*_w_/*M*_n_ distribution of polyethylene produced by Cp_2_TiMe_2_ precatalyst activated with B(C_6_F_5_)_3_ (see Table 3 in the article, respectively Table S9). Bimodal distribution is attributed to low thermal stability of the catalyst and consequent formation of multiple active species.

**Table S10.** Ethylene polymerisation results initiated by B(C_6_F_5_)_3_/Et_3_SiH systems. *^a^*

| Cat. | Polym.  medium | t  *(sec)* | T_2_  *(^0^C)* | PE  *(g)* | A | Si/  1000C^d^ | Si- terminated^e^  % | M_n_ | PDI |
| --- | --- | --- | --- | --- | --- | --- | --- | --- | --- |
| **5** | toluene | 500 | 30 | 0.810 | 130 |  |  | 147 700 | 2.81 |
| **5** *^b^* | toluene | 320 | 35 | 1.453 | 360 |  |  | 155 500 | 2.92 |
| **6** | toluene | 198 | 53 | 2.410 | 970 |  |  | 112 700 | 3.45 |
| **6** | CH_2_Cl_2_ | 103 | 54 | 2.033 | 1 580 |  |  | 75 000 | 3.43 |
| **7** | toluene | 197 | 44 | 1.934 | 790 |  |  | 71 300 | 4.83 |
| **7** | CH_2_Cl_2_ | 560 | 28 | 0.555 | 80 | 1.416 | 86 | 8 500 | 3.67 |
| **8** | toluene | 76 | 62 | 3.174 | 3 340 |  |  | 36 500 | 8.43 |
| **8** | CH_2_Cl_2_ | 317 | 46 | 1.915 | 480 | 2.624 | 84 | 4 500 | 3.14 |
| **8** *^c^* | CH_2_Cl_2_ | 55 | 44 | 1.256 | 1 830 | 2.441 | 64 | 3 700 | 3.26 |
| Cp_2_ZrMe_2_ | toluene | 35 | 74 | 2.757 | 6 300 |  |  | 21 000 | 6.14 |
| Cp_2_TiMe_2_ | toluene | 31 | 63 | 1.715 | 4 430 | 5.047 | 76 | 2 100 | 2.6 |
| Cp_2_TiMe_2_ | CH_2_Cl_2_ | 36 | 52 | 1.318 | 2 930 | 4.353 | 75 | 2 400 | 3.0 |
| Cp_2_TiCl_2_ | toluene | 1800 | *n.d.* | 0.074 | 3 | 0.19 | 11 | 8 200 | *n.d.* |
| Cp_2_TiCl_2_ | CH_2_Cl_2_ | 120 | *n.d.* | 1.570 | 1 050 | 2.88 | 92 | 4 500 | *n.d.* |

*^a^ Polymerisation conditions:* total volume 50 mL, catalyst dissolved in toluene, pressure 3 bar, T_1_ = starting temperature 25 ^o^C, T_2_ = final temperature before quenching, rpm 800, catalyst 15 μmol, ratio Et_3_SiH/cat. = 1 000; ratio B(C_6_F_5_)_3_/catalyst = 1.1. A = kg_PE_ (mol_cat_ bar h)^-1^. *^b^* Rpm 1 000. *^c^* 4.3 mL of CH_2_Cl_2_ was added to the catalyst solution in toluene and kept 24 h at room temperature under Ar.^d^ Number of Si atoms per 1000 C atoms in the polymer (determined by FT-IR). ^e^ Percentage of silyl-terminated polymer chains as determined by FT-IR.

**Figure S49.** FT-IR spectra (PE foils) of polymers obtained from ethylene polymerisation results initiated by B(C_6_F_5_)_3_/Et_3_SiH systems.

# 6 EPR experiments

## 6.1 Reaction with [Ph_3_C][B(C_6_F_5_)_4_]

The complexes **5**-**8** were placed together with equivalent amount (milligrams scale) of [Ph_3_C][B(C_6_F_5_)_4_] (TBT) into the EPR cuvette (equipped with a young valve or GL cap) in the glove box. Immediately before measurement, toluene was added under an argon atmosphere by syringe. The mixture reacted immediately to form a dark oily phase and a clear grey-green toluene solution. The cuvette was placed into the EPR spectrometer, and spectra were continuously collected. The presence of oily products in the resonance cavity was continuously checked.

**Figure S50.** X-band EPR spectra of **5**, in situ activation with [Ph_3_C][B(C_6_F_5_)_4_] (TBT) in toluene solution at room temperature after mixing.

**Figure S51.** X-band EPR spectra of **5**, in situ activation with TBT in toluene solution at room temperature after 24 hours from mixing.

**Figure S52.** X-band EPR spectra of **6**, in situ activation with TBT in toluene solution at room temperature after 1 minute from mixing.

**Figure S53.** X-band EPR spectra of **6**, in situ activation with TBT in toluene solution at room temperature after 24 hours from mixing.

**Figure S54.** X-band EPR spectra of **7**, in situ activation with TBT in toluene solution at room temperature after mixing.

**Figure S55.** X-band EPR spectra of **8**, in situ activation with TBT in toluene solution at room temperature after mixing.

**Figure S56.** X-band EPR spectra of **8**, in situ activation with TBT in mixture of toluene/THF at room temperature.

**Figure S57.** X-band EPR spectra of Cp_2_ZrMe_2_ in situ activation with TBT in toluene solution at room temperature after 15 minutes from mixing.

**Figure S58.** X-band EPR spectra of Cp_2_Zr(Me)(C_2_Ph) (**4**), in situ activation with TBT in toluene solution at room temperature after 10 minutes from mixing.

## 6.2 Reaction of 8 with B(C_6_F_5_)_3_ in toluene-*d*_8_ solution

**Figure S59.** X-band EPR spectra of reaction mixture **8** with B(C_6_F_5_)_3_ in toluene-*d*_8_ solution at room temperature.

# 7 NMR experiments

The NMR scale reactions were all performed under inert atmosphere as described above using appropriately dried solvents (including the deuterated ones). The sample solutions transferred into NMR tubes were degassed by freeze-thaw cycles and flame-sealed under vacuum to prevent unwanted contamination during the measurement.

**General procedure for the generation of cationic species.** In a similar way as for the EPR experiments, the studied complexes (typically 0.04 mmol) and an equimolar amount of the activating agent [Ph_3_C][B(C_6_F_5_)_4_] (TBT) or B(C_6_F_5_)_3_ were dissolved in 0.8 mL of toluene-*d*_8_ under vigorous shaking. The reaction mixtures immediately changed colour from yellow or orange (complexes **4**-**6**) or deep brown-green (**7** and **8**) to deep green or violet-blue (reaction of **5**) with a formation of viscous oily phase. The toluene soluble phase was transferred and analysed by NMR. The insoluble part was then dissolved in CDCl_3_ (optionally with a prior addition of few drops of THF and removal of all unreacted volatiles) and analysed by NMR spectroscopy. The THF addition allowed stabilisation of the cationic fragments.

In a separate experiment, *in situ* generation of the [Cp_2_TiCl(THF)]^+^ cation was performed by the following procedure utilising the chloride abstraction from Cp_2_TiCl_2_ by the silylium cation. Thus, Cp_2_TiCl_2_ (25 mg, 0.1 mmol) and TBT (92 mg, 0.1 mmol) were suspended in chlorobenzene (1 mL) and Et_3_SiH (48 µL, 0.3 mmol) was introduced and the mixture stirred until all the solids dissolved. The original yellow colour of TBT disappeared within 1 min and the crystalline Cp_2_TiCl_2_ then dissolved to give a green solution. After addition of few drops of THF all volatiles were removed under vacuum and the oily residue was dissolved in CDCl_3_ and analysed by NMR. The Cp signals in ^1^H at *δ* 6.72 ppm and in ^13^C at *δ* 122.87 ppm were tentatively ascribed to the [Cp_2_TiCl(THF)]^+^ cation (Figure S55a), while the same signals seem to be present in reactions of complexes **7** and **8** with TBT or B(C_6_F_5_)_3_.

Similarly, the formation of the cation [Cp*_2_ZrF(THF)]^+^ was described previously by some of us.^22^

**Figure S60.** ^1^H NMR spectrum (500.13 MHz, toluene-*d*_8_) of the reaction mixture (toluene-soluble part) of complex **4** with an equimolar amount of TBT.

**Figure S61.** ^1^H NMR spectra (500.13 MHz, toluene-*d*_8_) of the reaction mixtures (toluene-soluble part) of complexes **5**-**8** with an equimolar amount of TBT. Asterisk denotes signals of the mesitylene internal standard, # denotes the signals of Gomberg’s dimer (Ph_3_C(C_6_H_5_)=CPh_2_).

**Figure S62.** Identification of the ionic products after oxidation. ^1^H NMR spectra (500.13 MHz, CDCl_3_) of the reaction mixtures prepared as described above. Note the presence of signals of the suggested [Cp_2_TiCl(THF)]^+^ at *δ* 6.72 ppm in all reactions (marked by #) and Cp_2_TiCl_2_ (*δ* 6.59 ppm, marked by +). The anion MeB(C_6_F_5_)_3_^–^ (*δ* 0.59 ppm) is present in the reaction mixture d). Asterisk in the reaction mixture a) denotes the Ph_3_CH by-product of the reaction.

**Figure S63.** ^19^F NMR spectrum (470.55 MHz, CDCl_3_) of the reaction mixture b) in Figure S55 prepared as described above from complex **8** and TBT.

**Figure S64.** ^19^F NMR spectrum (470.55 MHz, CDCl_3_) of the reaction mixture d) in Figure S55 prepared as described above from complex **8** and B(C_6_F_5_)_3_ showing the formation of MeB(C_6_F_5_)_3_^–^ anion. Asterisk denotes the unreacted B(C_6_F_5_)_3_∙THF.

**Figure S65.** ^11^B NMR spectrum (160.46 MHz, CDCl_3_) of the reaction mixture d) in Figure S55 prepared as described above from complex **8** and B(C_6_F_5_)_3_ showing the formation of MeB(C_6_F_5_)_3_^–^ anion.

**Figure S66.** ^1^H NMR spectrum (500.13 MHz, CDCl_3_) of the CDCl_3_ solution of complex **8** after 15 min. The complex underwent complete decomposition, signals denoted # indicate the formation Cp_2_ZrClMe (identical to an authentic sample), + denotes the presence of Cp_2_TiCl_2_.

# 8 References

1. []	H. A. Hamwi, M. Rippke, K. Lindenau, A. Spannenberg, M. Lamac, F. Reiss, T. Beweries, *Chem. Eur. J.* **2025**, *31*, e202500857.
2. []	K. Lindenau, N. Jannsen, M. Rippke, H. Al Hamwi, C. Selle, H. J. Drexler, A. Spannenberg, M. Sawall, K. Neymeyr, D. Heller, F. Reiss, T. Beweries, *Catal. Sci. Technol.* **2021**, *11*, 4034–4050.
3. []	M. Fischer, L. Vincent-Heldt, M. Hillje, M. Schmidtmann, R. Beckhaus, *Dalton Trans.* **2020**, *49*, 2068–2072.
4. []	J. R. Nitschke, S. Zürcher, T. D. Tilley, *J.Am. Chem. Soc.* **2000**, *122*, 10345-10352.
5. []	G. R. Fulmer, A. J. M. Miller, N. H. Sherden, H. E. Gottlieb, A. Nudelman, B. M. Stoltz, J. E. Bercaw, K. I. Goldberg, *Organometallics* **2010**, *29*, 2176-2179.
6. []	G. M. Sheldrick, *Acta Cryst.,* *Sect. A: Found. Crystallogr.* **2008**, *64*, 112-122.
7. []	G. M. Sheldrick, *Acta Cryst.*, *Sect. C: Struct. Chem.* **2015**, *71*, 3-8.
8. []	H. Putz, K. Brandenburg, *Diamond-Crystal and Molecular Structure Visualization*, Crystal Impact-GbR, Kreuzherrenstr. 102, 53227, Bonn, Germany, **2018**.
9. []	S. Chen, J. Wang, H. Wang, *Materials & Design* **2016**, *90*, 84-90.
10. []	M. J. Frisch, G. W. Trucks, H. B. Schlegel, G. E. Scuseria, M. A. Robb, J. R. Cheeseman, G. Scalmani, V. Barone, G. A. Petersson, H. Nakatsuji, X. Li, M. Caricato, A. V. Marenich, J. Bloino, B. G. Janesko, R. Gomperts, B. Mennucci, H. P. Hratchian, J. V. Ortiz, A. F. Izmaylov, J. L. Sonnenberg, D. Williams-Young, F. Ding, F. Lipparini, F. Egidi, J. Goings, B. Peng, A. Petrone, T. Henderson, D. Ranasinghe, V. G. Zakrzewski, J. Gao, N. Rega, G. Zheng, W. Liang, M. Hada, M. Ehara, K. Toyota, R. Fukuda, J. Hasegawa, M. Ishida, T. Nakajima, Y. Honda, O. Kitao, H. Nakai, T. Vreven, K. Throssell, J. A., Jr. Montgomery, J. E. Peralta, F. Ogliaro, M. J. Bearpark, J. J. Heyd, E. N. Brothers, K. N. Kudin, V. N. Staroverov, T. A. Keith, R. Kobayashi, J. Normand, K. Raghavachari, A. P. Rendell, J. C. Burant, S. S. Iyengar, J. Tomasi, M. Cossi, J. M. Millam, M. Klene, C. Adamo, R. Cammi, J. W. Ochterski, R. L. Martin, K. Morokuma, O. Farkas, J. B. Foresman, D. J. Fox, *Gaussian 16, Rev. C.01*, Gaussian, Inc., Wallingford, CT, 2016.
11. []	a) E. D. Glendening, J. K. Badenhoop, A. E. Reed, J. E. Carpenter, J. A. Bohmann, C. M. Morales, C. R. Landis, F. Weinhold, Theoretical Chemistry Institute, University of Wisconsin, Madison, **2013**; b) J. E. Carpenter and F. Weinhold, *J. Mol. Struct.: THEOCHEM* **1988**, *169*, 41-62; c) F. Weinhold, J. E. Carpenter, in *The structure of small molecules and ions*, Springer, **1988**, 227-236; d) F. Weinhold, C. R. Landis, *Valency and bonding: a natural bond orbital donor-acceptor perspective*, Cambridge University Press, **2005**.
12. []	a) A. D. Becke, *Phys. Rev. A* **1988**, *38*, 3098-3100; b) J. P. Perdew, *Phys. Rev. B* **1986**, *33*, 8822-8824.
13. []	a) S. H. Vosko, L. Wilk, M. Nusair, *Can. J. Phys.* **1980**, *58*, 1200-1211; b) C. Lee, W. Yang, R. G. Parr, *Phys. Rev. B* **1988**, *37*, 785-789; c) B. Miehlich, A. Savin, H. Stoll, H. Preuss, *Chem. Phys. Lett.* **1989**, *157*, 200-206; d) A. D. Becke, *J. Chem. Phys.* **1993**, *98*, 5648-5652.
14. []	F. Weigend, R. Ahlrichs, *Phys. Chem. Chem. Phys.* **2005**, *7*, 3297-3305.
15. []	a) S. Grimme, J. Antony, S. Ehrlich, H. Krieg, *J. Chem. Phys.* **2010**, *132*, 154104; b) S. Grimme, S. Ehrlich, L. Goerigk, *J. Comput. Chem.* **2011**, *32*, 1456-1465.
16. []	T. Lu, F. Chen, *J. Comput. Chem.* **2012**, *33*, 580-592.
17. []	R. Dennington, T. A. Keith, J. M. Millam, GaussView, version 6.0. 16. *Semichem Inc. Shawnee Mission KS*, **2016**.
18. []	Mercury: <http://www.ccdc.cam.ac.uk/mercury/>
19. []	P. Pracht, S. Grimme, C. Bannwarth, F. Bohle, S. Ehlert, G. Feldmann, J. Gorges, M. Müller, T. Neudecker, C. Plett, S. Spicher, W. Steinbach, P. A. Wesołowski, F. Zeller, *J. Chem. Phys.*, **2024**, *160*, 114110-114128.
20. []	C. Bannwarth, S. Ehlert, S. Grimme, *J. Chem. Theory Comput.* **2019**, *15*, 1652–1671.
21. []	L. Jia, X. M. Yang, A. Ishihara, T. J. Marks, *Organometallics* **1995**, *14*, 3135–3137.
22. []	V. Varga, M. Lamač, M. Horáček, R. Gyepes, J. Pinkas, *Dalton Trans.* **2016**, *45*, 10146–10150.
23. []	a) R. Blom, A. Follestad, O. Noel, *J. Mol. Catal.* **1994**, *91*, 237–249; b) K. Thorshaug, J. A. Støvneng, E. Rytter, M. Ystenes, *Macromolecules* **1998**, *31*, 7149-7165.
